# Both movements and breeding performance are affected by individual experience in the Bonelli's eagle *Aquila fasciata*

**DOI:** 10.1002/ece3.70081

**Published:** 2024-07-24

**Authors:** Lise Viollat, Alexandre Millon, Cécile Ponchon, Alain Ravayrol, Thibaut Couturier, Aurélien Besnard

**Affiliations:** ^1^ CEFE, Univ Montpellier, CNRS, EPHE‐PSL University, IRD Montpellier France; ^2^ Aix Marseille Université, Institut Méditerranéen Biodiversité et Ecologie Marine et Continentale, CNRS, IRD, Avignon Université, Technopôle Arbois‐Méditerranée Aix‐en‐Provence France; ^3^ CEN PACA Saint‐Martin de Crau France; ^4^ La Salsepareille Clermont l'Hérault France

**Keywords:** bird of prey, demography, flight, GPS tracking, long‐lived, movement behaviours, territory

## Abstract

Movement is a key behaviour to better understand how individuals respond to their environment. Movement behaviours are affected by both extrinsic factors that individuals face, such as weather conditions, and intrinsic factors, such as sex and experience. Because of the energy costs it entails, movement behaviours can have direct consequences on an individual's demographic parameters—and ultimately on population dynamics. However, the relationship between extrinsic factors, intrinsic factors, daily movement behaviour and demographic parameters such as breeding performance is poorly known, in particular for central place forager territorial species. We investigated here the link between movement behaviours and breeding performance of the French population of Bonelli's eagle (*Aquila fasciata*), a territorial and sedentary long‐lived raptor, and how this link may depend on extrinsic and intrinsic factors. By using data from annual monitoring of breeding performance for the population and GPS tracking of 48 individuals (26 males and 22 females), we found that the breeding performance of this population was mainly driven by whether a new individual was recruited into the territory, and only slightly by weather conditions. Movement behaviours (proportion of time in flight, range of movement and straightness of trajectories) showed large between‐individual variation. Those behaviours were related with weather conditions (wind and rainfall) at a daily scale, as well as with individual's experience. We found only one significant correlation between movements and breeding performance: male Bonelli's eagles spending more time flying during chick‐rearing phase had lower productivity. Movement behaviours and breeding performance were also indirectly linked through individual's experience, with more experienced birds having better breeding success and a shorter range of movement and spent less time in flight. This suggests that experienced individuals progressively acquire knowledge of their breeding territory, are more efficient in finding prey, and adapt their foraging strategies to weather conditions to minimise energy costs, allowing them higher breeding performance.

## Graphical abstract

Using jointly demographic data and GPS tracking on the same population, we were able to investigate the indirect and direct relationships between the movement behaviours, intrinsic and extrinsic factors and the breeding success of a long‐lived territorial birds of prey, the Bonelli's eagle. Our results suggest that movements of Bonelli's eagles were strongly driven by experience and by daily weather conditions, while breeding success was mainly driven by an individual's experience of the breeding site and only less so by seasonal weather conditions. This suggests that long‐lived species might be able to avoid strong negative effects of weather conditions on their breeding success by adapting their daily movements and foraging strategies.

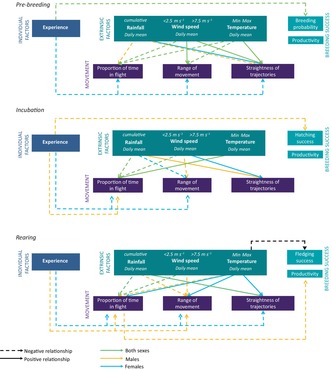

## 1 INTRODUCTION

Bird population dynamics are influenced by extrinsic factors, such as weather conditions and food availability that individuals experience throughout their life (Newton, 1998). Individuals also differ greatly from each other in terms of intrinsic factors such as sex, age, personality or size, and thus express different behaviours in response to the local extrinsic factors they experience (Jenouvrier et al., 2015). This individual heterogeneity in intrinsic factors and behaviours plays an important role in demographic parameter variation (Cam et al., 2013; Gimenez et al., 2018) and in turn affects population dynamics (Bjørnstad & Hansen, 1994; Kendall et al., 2011; Lomnicki, 1978). To better predict vertebrate populations dynamics, it is thus crucial to assess the interplay between intrinsic and extrinsic factors on population dynamics (Lande et al., 2003).

A key behaviour of birds is movement, allowing them to acquire resources at a larger scale compared to non‐flying animals (Shepard & Lambertucci, 2013) and adapt to their local habitat and weather conditions. Individuals life history involves movement at different temporal and spatial scales, ranging from daily foraging movements inside a territory, to dispersal between territories or between populations, or even at a transcontinental scale for migration (Mueller & Fagan, 2008; Nathan et al., 2008). Each movement implies a trade‐off between the benefit it procures (acquisition of resources, protection against predators, reproduction) and the energetical cost it entails (McLoughlin et al., 2006; Morales et al., 2010). As a result of this trade‐off, movements may therefore have direct consequences on the survival and breeding performance of the individuals (Nathan et al., 2008; Pennycuick, 1989), and thus on population dynamics (Penteriani & Delgado, 2009). Bird flight, in particular, implies high energetical costs for individuals (Dussault et al., 2012; McLoughlin et al., 2006; Morales et al., 2010; Pennycuick, 1989). Investigating how birds move can be a powerful tool to understand the complex interplay between intrinsic, extrinsic factors and avian demography.

While movements are mostly influenced by an individual's ability to move and motivation (to breed, to feed, to defend territory, Nathan et al., 2008), they can also be affected by sex or experience. For instance, it has been shown that a bird's migratory performance improves with age and experience (Miller et al., 2016) as it accumulates information and knowledge about its environment (Fagan et al., 2013), improves its navigating and orientation skills (Mueller et al., 2013; Thorup et al., 2007) and learns how to minimise energy costs (Maransky & Bildstein, 2001). Different foraging behaviours between sexes has also been observed in several bird species with sexual size dimorphism (Lewis et al., 2002). Individual movements are also influenced by external conditions to adapt behaviours to changes in the environment such as local weather conditions (Allen & Singh, 2016; Morales et al., 2010; Nathan et al., 2008; Pyke, 2019). Strong winds or rainfall may affect flight manoeuvrability, or even prevent birds from flying (Amélineau et al., 2014). The flight of large birds such as raptors can highly depend on weather conditions. For example, the presence of thermal uplift favours soaring (Duerr et al., 2015; Katzner et al., 2012; Klaassen et al., 2010). Weather conditions might also change a bird's flight strategies and the way it forages (Cecere et al., 2020; Hernández‐Pliego et al., 2017; Sergio, 2003). By affecting the movement behaviours of individuals, extrinsic factors can ultimately affect demographic parameters. For example, intense rainfall during breeding season could negatively affect the breeding success of birds (Anctil et al., 2014; Kostrzewa & Kostrzewa, 1990), possibly linked to reduced bird flight and foraging capacity. Poor weather conditions for flying may increase the energy expenditure (Pennycuick, 1989), by forcing birds to do more flapping flight or increasing foraging time for example, and may negatively impact their breeding success.

However, the potential effects of intrinsic and extrinsic factors on the daily movements of territorial bird species, as well as the links between daily movements behaviours and breeding performance, are still poorly known, despite the fact these may have major consequences on breeding success and survival. For a long time, demographic studies and movement studies relied on very different methodological approaches: the former has involved general monitoring of a large number of marked individuals over the long term to study survival and fecundity (capture‐mark‐recapture approach), whereas the latter has involved detailed monitoring (several locations recorded per day) of a limited number of individuals over shorter periods of time. Now, technological advances and decreased costs of satellite telemetry techniques allow access to data on the daily movement behaviours of a large number of individuals over several years (Cooke et al., 2004; Rutz & Hays, 2009; Seegar et al., 1996; Wilmers et al., 2015), allowing an investigation of links between movement behaviours and demographic parameters.

Using long‐term GPS tracking, our aim was to investigate the links between movement behaviours and breeding performance of the French population of Bonelli's eagle (*Aquila fasciata*) and how these links depend on extrinsic and intrinsic factors. Bonelli's eagle is a long‐lived and territorial raptor, occupying mostly open environments such as scrubland and limestone cliffs (Del Hoyo et al., 1994). Adults are highly territorial and sedentary, with strong site fidelity (Hernández‐Matías et al., 2015). They live in pairs in a well‐defined territory that is stable over time, that they use for all their daily activities, such as foraging, resting, breeding and rearing young (Börger et al., 2008; Burt, 1943). A long‐term conservation programme dedicated to the population has been in place since 1990 in France, using ringing to assess survival and breeding performance. The resulting monitoring has allowed good knowledge about individual characteristics such as sex, age, year of recruitment or years on the breeding site (Chevallier et al., 2015; Lieury et al., 2016). In the framework of this programme, 48 adult individuals have been equipped with GPS trackers, providing a large amount of data on daily movements, allowing knowledge of their territory and habitat use. Given this access to both demographic and movement data on specific individuals, the relationships between demography and movement behaviours in this territorial species can be explored. As a long‐lived species, Bonelli's eagles are likely to favour their own survival over reproduction (Hamel et al., 2010; Mourocq et al., 2016; Stearns, 1992) and would be likely to adapt their behaviours in response to extrinsic conditions, to ensure their survival (Bradley et al., 2000; Covas et al., 2004; Shaw & Levin, 2013). Furthermore, as flight is particularly costly in energy (Pennycuick, 1989), it could have strong effects on an individual's breeding performance by affecting body condition. We thus decided to specifically focus on breeding performance (breeding probability, hatching success, fledging success and productivity), and on movement during the breeding season. We identified three key phases during the breeding season when males and females might display different breeding behaviours and strategies (Martínez et al., 2020) and analysed these separately: (1) the pre‐breeding phase when Bonelli's eagles perform courtship, mate and prepare the nest; (2) the incubation phase when the female mostly stays on the nest while the male forages for food; and (3) the rearing phase, from the hatching of the chicks to fledging, when both parents feed and take care of chicks in the nest, protecting them from predation and bad weather conditions. We expected to find large differences in movements throughout the breeding season due to these different behaviours—particularly between males and females during the incubation, as Bonelli's eagles exhibit sex‐specific parental roles, with females ensuring incubation and taking care of young chicks at the nest, while males contribute more to food provisioning (López‐López et al., 2022; Martínez et al., 2020).

Our approach involved two main steps. First, we investigated potential indirect links between movement and breeding performance by testing the effect of local weather conditions (rainfall, wind speed, temperature) and intrinsic factors (sex and experience) on both. Secondly, we tested the direct effects of movement on breeding performance for each breeding phase and sex. We expected strong effects of an individual's experience on both movement and breeding performance, as an individual's strategies might be more effective with increased knowledge of its breeding site (Daunt et al., 2007; Newton, 1989; Pärt, 1997). We also expected strong effects of daily local weather conditions on an individual's movements, particularly rainfall and wind speed, as flight might be constrained by intense wind or rainfall (Lehikoinen et al., 2009; McDonald et al., 2004; Penteriani, 1997; Sergio, 2003). We expected that higher temperatures would increase movement due to the occurrence of thermal uplift (Duerr et al., 2015; Katzner et al., 2012; Shamoun‐Baranes et al., 2003). Concerning the effects of local weather conditions on breeding performance, we expected negative effects from bad weather conditions (heavy rainfall, strong winds, low or high temperature), as these might have direct consequences on the body condition of the chicks or the parents due to thermoregulation needs, and/or by preventing parents from foraging. We hypothesised that a higher proportion of time in flight, a larger range of movement, and straighter trajectories would negatively impact breeding performance, especially during the rearing phase, as it could reflect costlier movements or difficulty in foraging.

## 2 MATERIALS AND METHODS

### 2.1 Bonelli's eagle breeding

The Bonelli's eagle reaches sexual maturity between the age of 2 and 5 (Hernández‐Matías et al., 2010; López‐Peinado & López‐López, 2023). After an erratic phase, individuals settle in a territory in pairs, usually replacing an individual that has disappeared from an existing pair, forcing an individual to leave its territory (take‐over) or colonising a new territory together with a mate (Hernández‐Matías et al., 2011). Both partners are faithful for life (Hernández‐Matías et al., 2015). The breeding season can be split into three phases (pre‐breeding, incubation and rearing) in which individuals express specific behaviours. Females invest more effort in incubation and taking care of the eggs and chicks in the nest, while males contribute more to food provisioning (Martínez et al., 2020). During the pre‐breeding (from the start of winter), the pair performs courtship and builds and/or restores nesting areas. Mating takes place from January to March. The female lays one or two eggs (rarely 3, Gil‐Sánchez et al., 2004), between February and March, marking the beginning of the incubation phase, which usually lasts 37–41 days (Arroyo et al., 1995 in López‐Peinado & López‐López, 2023). During incubation, the female usually stays on the nest, while the male hunts (López‐López et al., 2022). Eggs hatch in early spring, between April and May. During the rearing phase, the male generally hunts, while the female feeds the chicks and protects them from the sun. Nest attendance of females decreases progressively over the rearing phase, and both parents adjust their provisioning effort to nestling needs (López‐López et al., 2022; Martínez et al., 2020). Chicks fledge on between 55 and 65 days after the hatching, between late May and early July, and leave their parents' territory between 77 and 113 days after fledging to begin a dispersal phase (Ferguson‐Lees & Christie, 2001; Real et al., 1998). The timing of the different breeding phases presented here concerns the French population (Burger et al., 2013), and differences may be observed with other populations.

### 2.2 Demographic data

The French population of Bonelli's eagle is distributed across the south west of the French Mediterranean area, from 42°63′ N to 44°61′ N and from 2°52′ E to 6°35′ E (Hernández‐Matías et al., 2013; Lieury et al., 2016). It has been intensively monitored since 1990. This involves ringing chicks in the nest, ring resighting and surveying reproduction. All known breeding pairs (between 22 and 46 depending on the year) were monitored each year (Lieury et al., 2016), via repeated visits in January–July (minimum three visits but typically 6–10) which provided information about the territory occupancy and nesting sites, individuals identity (if ringed), if a clutch was laid, if eggs had hatched, the number of nestlings and the number of fledged chicks. Observations were made with a telescope at distances from nests (typically >300 m) to avoid disturbance. Between 12 and 39 nestlings (average: 25 ± 7) have been ringed annually in the study population since 1990 (*n* = 840 between 1990 and 2022). Once they were 35–45 days old, nestlings were fitted with a conventional metal ring and an alphanumerically coded coloured metal ring that can be read using a telescope at a distance up to 200 m. Ringing was conducted by retrieved the chicks from the nest by experienced climbers to minimise the time spent away from the nest. The ringing operation lasted about 1 h. Sex of chicks was established during ringing according to their body mass and width of the tarsi. Bonelli's eagles are sexually dimorphic (García et al., 2013), females being substantially larger than males (Hernández‐Matías et al., 2011). Territorial individuals have an estimated resighting probability of 63% (Lieury et al., 2016).

### 2.3 Movement data

In addition to the ringing programme, between 2009 and 2021, 48 adult individuals (22 females and 26 males) from 33 different breeding sites were captured and equipped with GPS tags (from different manufacturers, see Appendix S6) powered by solar panels. Individuals were captured using a bait and a remotely activated folding net. The trap was only activated once the targeted individual was inside the catching area. A hood was then placed over the bird eyes to reduce stress and the talons were bandaged to prevent injuries. Captures took place outside the breeding season. The tag was fitted to the bird's back with using a backpack Teflon harness following the Garcelon harnessing method (García et al., 2021). No marks, injuries, feather or skin abrasion that could have been caused by the harness or the transmitter has ever been observed in large raptors using this harnessing method (García et al., 2021). The transmitter and harness represented 1.2%–3.5% of total body weight. The bird was handled for a total of about 20 min. Tagged individuals were then surveyed for an average of 734 days (ranging from 36 to 2037 days) over this period, the duration of the tracking depending on the GPS tag brand and the survival of individual. The datasets for each individual were cleaned to remove outliers, especially inaccurate locations or impossible dates, following the recommendations of Gupte et al. (2021). Known locations error specific to brand and model of GPS transmitter were corrected and cleaned. We deleted data with a horizontal dilution of precision (Hdop) >10 or a satellite number <3 (Silva et al., 2017). Depending on the model and manufacturer, we did not have the same number of locations between GPS tags. Furthermore, for a GPS tag, we did not necessarily have regular time intervals between each point, even within a day, due to GPS settings to conserve battery life issues and our partners' different data collection objectives. We kept a minimum interval of 15 min between each GPS location as it was the most common interval available in the GPS data. We accounted for the variation of the number of locations on the metrics of movement by including it as a covariate in our models. To avoid as much as possible bias in the estimates of our metrics related to movements, we removed individual‐days with less than 10 locations, considering that the proportion of time in flight would suffer strong random variations with such a sample size.

To qualify the movement behaviours of a Bonelli's eagle at a daily scale inside its territory, we estimated three proxies. (i) The range of movement (in m), computed by determining the median distance between all locations recorded in a single day and the first location, presumed to be the individual's resting point during the night. Using the first location as a reference point, this range provides a proxy of individual movements that can be used to compare individuals, but does not reflect real movements made by individuals. To ensure the accuracy of the analysis, we excluded days when the initial location fell outside the time window of 30 min before and 2 h after sunrise. This time frame corresponds to the time when Bonelli's eagles typically become active. The distance was calculated using the function distGeo from the package geosphere (Hijmans et al., 2022). We used the median distance rather than the mean distance, as the median is less sensitive to extreme values, and is thus more relevant to compare the daily range of movement individuals. (ii) The straightness of trajectories performed during the day (calculated with the function TrajStraighness of the package *trajr*, McLean & Skowron Volponi, 2018). The straightness of trajectories was calculated as *D*/*L*, where *D* was the straight‐line distance between the first point and the last point of the trajectory, and *L* the distance through all points of the trajectory (Batschelet, 1981). The closer to 1, the straighter the trajectory. A sinuous trajectory (close to 0) may reflect territory exploration or foraging behaviours, while a value of 1 may reflect transit movements between two locations. (iii) The proportion of time in flight, calculated as the number of locations in flight over the total number of GPS locations for 1 day. A bird was considered to be in flight when its speed exceeded 3 m s^−1^. An individual is in flight when exploring its territory, during certain pair‐bonding movements (feasting), during transit from point A to point B inside the territory (from the nesting area to a hunting spot, for example) or during foraging activities.

### 2.4 Effect of intrinsic and extrinsic factors on breeding performance

We studied three breeding parameters to gain a better understanding of the breeding performance of Bonelli's eagle: breeding probability (pair having laid among the pairs present on a breeding site), hatching success (pair having at least one hatching among the pairs having laid eggs), and fledging success (pair having fledged at least one young among pairs having hatched at least one egg). The number of successful and unsuccessful events for each breeding parameter is displayed in Appendix S1. We fitted generalised linear mixed models (GLMM) with a binomial distribution for all breeding parameters to model the relationship between the breeding parameters with intrinsic factors and local weather variables. These relationships were studied for breeding events recorded in the French population of Bonelli's eagle between 2009 and 2022, i.e. 455 breeding events on 39 breeding sites. We included a random effect for the breeding site to consider inter‐breeding site heterogeneity and repeated measurements. We considered the experience of an individual on its breeding site as an intrinsic factor. As we did not have data to test the number of years spent on the breeding site for all individuals, we used recruitment as a proxy for breeding experience on their breeding site. We then distinguished between individuals that had just recruited and had no previous breeding experience and individuals who had already bred on their site. Recruitment was included in the models as a categorical variable with four levels for breeding probability (0 = no recruitment on the breeding site, F = recruitment of a female, M = recruitment of a male, and MF = recruitment of both individuals on a breeding site) and three levels for hatching and fledging success (as we had no event with recruitment of both male and female in these datasets). The year of recruitment coincides with the first known breeding event of individuals with no previous experience on the breeding site, with the uncertainty that the individual may have been recruited in a previous breeding season but not detected. The years with uncertainties about a potential recruitment have been removed (<1.5% of the original dataset). To assess the effect of local weather conditions, we included in the model the cumulative rainfall (in mm) for the phase, the minimum and maximum mean daily temperature observed for the phase, and the number of days with wind speed above 7.5 m s^−1^ and below 2.5 m s^−1^. The local weather data were extracted for each phase (pre‐breeding, incubation and rearing) from the SAFRAN dataset (Méteo France), at an 8 × 8 km resolution. The wind thresholds of 7.5 and 2.5 m s^−1^ were chosen based on our results of the effect of wind on our metrics related to daily movement behaviour (see Section 3 and Appendices S4 and S5). As we did not have the home range for sites where no individuals were equipped with GPS tags, we took the daily data within a 70 km^2^ disc around the centroid of known nests (Bonelli's eagles have typically 2–3 nests within their territory) for each territory, to represent the potential area that individuals could use and therefore the area where they could be subjected to weather conditions (70 km^2^ being the average home range during the breeding season for this population, unpublished results). For each breeding parameter, we chose to study the effects of weather conditions only during the phase preceding the event (weather conditions during pre‐breeding for breeding probability, during incubation for hatching success, and during rearing for fledging success). The distributions of the weather variables used in this study are available in Appendix S2. Intrinsic factors and extrinsic factors were considered as additive effects in the models.

### 2.5 Effect of intrinsic and extrinsic factors on movement behaviours

First, we studied if movement behaviours differ between breeding phases (pre‐breeding, incubation and rearing) and sex. We averaged the daily data of movement behaviours (proportion of time in flight, range of movement and straightness of trajectory) for GPS‐equipped individuals for each phase (19 females and 24 males from 32 breeding sites, corresponding to individuals equipped with GPS with available data during the breeding season). We then fitted linear mixed models with sex and phases as categorical variables in interaction to assess their effect on the mean value of movement behaviours averaged by phase, with an individual random effect to consider individual heterogeneity and repeated measurements. To assess heterogeneity between individual behaviours, we calculated the coefficient of variation (i.e. the ratio of the standard deviation to the mean) for each movement behaviour proxy averaged by phase and sexes separately.

Secondly, we fitted linear mixed models to estimate the relationships between the three metrics related to movement behaviours (proportion of time in flight, range of movement [log‐transformed] and straightness [logit‐transformed]) of GPS‐equipped individuals (19 females and 24 males from 32 breeding sites, for which data were available during the whole breeding season) with intrinsic factors and local weather conditions at a daily scale. We used LMMs (gaussian distribution) for the range of movement and straightness of trajectories, and a GLMM with a binomial distribution for the daily proportion of time in flight (the number of locations in flight [<3 m s^−1^] vs. not in flight). We fitted a model for each of the three defined phases (pre‐breeding, incubation and rearing) for each sex. We included an individual random effect (a unique ID for each bird) in the models to consider individual heterogeneity and repeated measurements. The following fixed effects were included in the models to assess their effects on intrinsic factors and local weather conditions on daily movement behaviours: the number of years that an individual has spent on its breeding site (used as a proxy of the individual's breeding experience, see Appendix S1), the daily rainfall (mm), the mean daily wind speed (m s^−1^), and the mean daily temperature (°C) inside each home range (kernel 95% defined with the R package *adehabitatHR*; Calenge, 2006 of the individuals equipped with a GPS tag). We extracted daily local weather data from the SAFRAN dataset (Méteo France), at an 8 × 8 km resolution. When the home range crossed several SAFRAN cells, we averaged the data available on the different cells for 1 day. The distributions of the weather variables used in this study are available in Appendix S2. We added quadratic terms for wind speed, on the hypothesis that there might be optimal wind speed conditions that allow individuals to browse longer distances, pass more time in flight and make active searching movements with less energy expense. To avoid potential bias from over‐ or underestimates due to the number of GPS locations, we also assessed the effects of this number on the range of movement and on straightness, as the number of locations was not standardised for each day and for each individual. The number of GPS locations was included in the calculation of the proportion of time in flight, so was not added in the models. Intrinsic factors and extrinsic factors were considered as additive effects in the models.

### 2.6 Relationship between movement behaviours and productivity

We tested the effect of the three metrics related to movement behaviours (proportion of time in flight, range of movement and straightness) on productivity for GPS‐tagged individuals (65 breeding events on 26 sites). The productivity was defined as the number of fledging chicks (0, 1 or 2). The number of breeding events with 0, 1 or 2 chicks are displayed in Appendix S1. Only data from individuals for whom we had GPS locations throughout at least one complete breeding season (pre‐breeding, incubation, and rearing phases) were used in the analysis (see Appendix S6). To consider the hypothesis that Bonelli's eagles show different behaviours throughout the breeding season with differences between sexes and phases, we fitted several LMMs with sexes separated, and movement behaviours averaged for the pre‐breeding, incubation and rearing phase. As the number of fledging chicks in our dataset do not exceed two and following the recommendation of McDonald and White (2010) that compared regression model for small counts with low variance, typical of productivity data, we considered an LMM with a Gaussian distribution instead of a Poisson distribution, the later may potentially produce a number of fledging higher than two.

The different models fitted in this study with their covariables are summarised in the Table 1. For each model, all continuous explanatory variables were standardised as $Z_{i}=\left(X_{i}-\bar{X}\right)/\sigma$ (with *Z* the standardised variable, *X* the original variable and *σ* its standard deviation) to allow the comparison of the different effect sizes among each other. Correlations between variables (Pearson correlation coefficient > .6) were verified. In order to be able to compare the different models together and the effect size of the different variables, we did not perform model selection. Parameters were considered significant when the *p*‐value was below .05 (*p* < .05). We also discussed results with *p* < .1, as showing a potential but marginal effect. All models were fitted in program R (version 4.1.2) with library *glmmTMB* (Brooks et al., 2017). All estimated values are shown in Appendix S5. We specified all estimates with their 95% confident interval in brackets.

**TABLE 1:** Mean, standard deviation (SD), minimum (Min), maximum (Max) and coefficient of variation (expressed as percentages, CV%) for three movement behaviours (proportion of time in flight, range of movement and straightness of trajectories) in the Bonelli's eagle according to breeding phases and sex.

	Sex	Proportion of time in flight					Range of movement					Straightness of trajectories				
		Mean	SD	Min	Max	CV%	Mean	SD	Min	Max	CV%	Mean	SD	Min	Max	CV%
All periods	M	0.21	0.05	0.04	0.22	29	1945	708	1004	3912	38	0.1	0.05	0.04	0.22	43
	F	0.19	0.07	0.04	0.23	51	1737	602	856.3	2996	38	0.11	0.06	0.04	0.23	46
Pre‐breeding	M	0.17	0.05	0.05	0.38	24	2032	903	957.4	5064.1	44	0.15	0.09	0.05	0.38	59
	F	0.15	0.08	0.05	0.32	66	1817	847	624.8	3580.6	45	0.16	0.09	0.05	0.32	50
Incubation	M	0.26	0.06	0.01	0.13	23	1892	854	696.2	3689	44	0.06	0.03	0.02	0.13	69
	F	0.19	0.13	0.004	0.35	39	1088	933	36.49	2866.27	87	0.09	0.09	0.004	0.35	100
Rearing	M	0.27	0.07	0.01	0.2	73	1673	767	674.4	3713.2	46	0.06	0.05	0.01	0.20	73
	F	0.24	0.11	0.01	0.13	67	1298	888	282.7	3735.9	66	0.06	0.04	0.01	0.13	67

## 3 RESULTS

### 3.1 Effects of intrinsic and extrinsic factors on breeding performance

Between 2009 and 2022 in the French population of Bonelli's eagle, we observed, on average, that territorial pairs have a probability of 0.63 [0.57, 0.69] of having at least one fledgling. Territorial pairs had a probability of 0.85 [0.81, 0.88] to lay a clutch (breeding probability). Among the pairs having a clutch, the probability of having at least one hatching was 0.82 [0.78, 0.86] (hatching success). Among the pairs having at least one hatchling, the probability of having at least one fledgling was 0.90 [0.87, 0.93] (fledging success). The average number of fledged chicks among territorial pairs (i.e. the productivity) was 0.99 [0.91, 1.06].

**FIGURE 1:**
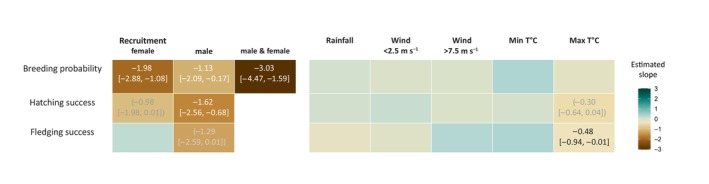
Summary of the effect of intrinsic factors (recruitment of a female, a male, or both on a breeding site) and local weather conditions (rainfall, number of days with wind speed above 7.5 m s^−1^ or below 2.5 m s^−1^, minimum and maximum temperature [min T°C and max T°C]) on breeding probability, hatching and fledging success of Bonelli's eagles, from GLMM models. Tile colour refers to the slope estimated by the models. Estimated coefficients and 95% confidence intervals are reported when *p* < .1. Grey coefficients between parentheses indicate a marginally significant effect (.1 < *p* < .05). The variables were standardised in the models so the estimated slopes can be compared with each other.

Concerning intrinsic factors (Figure 1), we found negative relationships between the recruitment of new individuals on a breeding site and breeding performance. Breeding probability was 0.95 when there was no new recruitment, decreasing to 0.85 if a male recruited on the breeding site, to 0.71 when a new female was recruited, and to 0.46 if both individuals recruited on a breeding site. We also found a negative relationship between the recruitment of a male on a breeding site and hatching success, as well as a negative tendency if a female recruited (−0.98 [−1.98, 0.01], *p* = .05). Male recruitment did not affect fledging success, but newly recruited females tended to obtain lower fledging success (−1.29 [−2.59, 0.01], *p* = .05; Figure 1). Regarding local weather conditions, we found negative relationships between maximum temperature experienced during rearing and incubation and both fledging and hatching success, though the latter was only marginally significant (−0.30 [−0.64, 0.04], *p* = .08). We did not detect any correlation between rainfall or wind conditions and breeding performance (Figure 1).

### 3.2 Effects of intrinsic and extrinsic factors on movement behaviours

During the breeding season (all three phases combined), the 48 GPS‐tagged Bonelli's eagles spent on average 20 ± 0.1% of the daytime in flight, had an average range of movement of 1859 ± 665 m, and flew in trajectories with a straightness coefficient of 0.10 ± 0.05.

**FIGURE 2:**
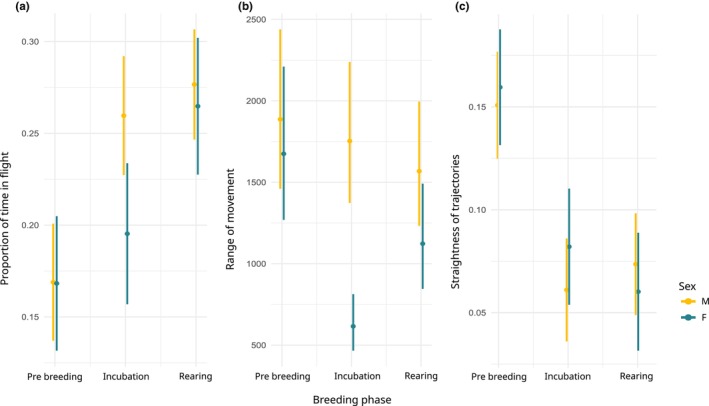
Estimated values and 95% confidence interval of movement behaviour of the Bonelli's eagle: (a) proportion of time in flight, (b) range of movement and (c) straightness of trajectories according to breeding phases and sexes.

Movement behaviours varied between phases and between sexes (Table 1, Figure 2). Effect sizes are available in Appendix S5. As expected, the proportion of time in flight of females was smaller during incubation than the one of males (30% higher). However, we did not find any significant difference between sexes during the pre‐breeding or rearing. For both sexes, the proportion of time in flight was higher (52% higher for females and 65% for males) during rearing than during pre‐breeding. The range of movement of males was mostly stable throughout the breeding seasons, with a slight but not significant decrease at the end of the season. During the pre‐breeding phase, males and females did not show significant differences in their range of movement. The range of movement of females during incubation was 63% smaller than during pre‐breeding and 65% smaller than the range of movement of males during incubation. We did not observe a significant difference between the mean daily straightness of males and females, regardless of the phase. However, the daily straightness was lower during incubation (50% for females and 60% for males) and rearing (63% for females and 53% for males) compared to pre‐breeding.

We observed large differences in movement behaviours between individuals (Table 1, Appendix S3), with coefficients of variation above 20% for all variables (Table 1). This inter‐individual variation in movement behaviours was particularly high for females during incubation and rearing.

**FIGURE 3:**
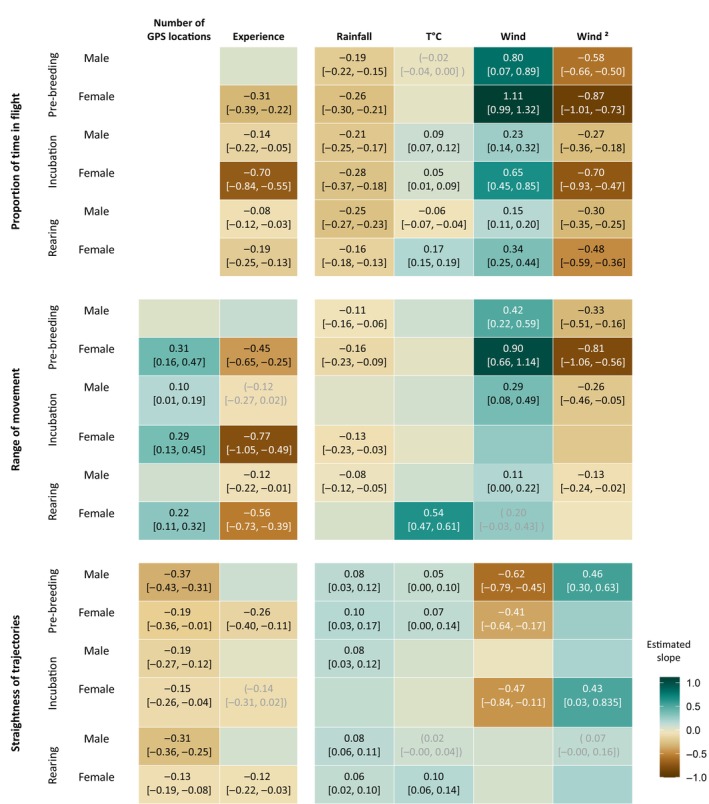
Estimated slopes for the effect of number of GPS locations, individual experience (number of years spent on a breeding site since recruitment) and local weather conditions (rainfall, temperature [T°C], and wind speed [Wind and Wind^2^]) on the proportion of time in flight, range of movement and straightness of trajectories of the French population of Bonelli's eagle, depending on the breeding phase (pre‐breeding, incubation and rearing) and sex. Tile colour indicates the slope estimated by GLMM models (1 model per sex and per period for the three movement behaviours) with X as a random factor. Estimated coefficients and 95% confidence intervals are shown when *p* < .1. Grey coefficients between parentheses indicate a marginally significant effect (.1 < *p* < .05). The variables were standardised in the models so the estimated slopes can be compared with each other.

More experienced females displayed an increased proportion of time in flight whatever the phase (Figure 3). The same was true for experienced males during the incubation and rearing compared with individuals that had spent fewer years on a breeding site. Experienced females also showed reduced straightness compared to recently recruited females during all phases (marginally significant during incubation; −0.14 [−0.31, −0.11], *p* = .09). We did not find any correlation between the straightness of trajectories and the experience of males, whatever the phase. An individual's experience showed contrasting correlation with the range of movement: experienced females during pre‐breeding and incubation, and experienced males during rearing, showed a larger range of movement compared to less experienced individuals, whereas experienced females showed a smaller range during rearing. We also found a marginally significant negative relationship between the experience of males and the range of movement during incubation (−0.12 [−0.27, 0.02], *p =* .09).

Local weather conditions affected the daily movements of Bonelli's eagles differently depending on phase and sex (Figure 3). Whatever the phase and for both sexes, the proportion of time spent in flight was negatively correlated with rainfall. We also found a negative correlation between rainfall and the range of movement of males and females during pre‐breeding, as well as females during incubation and males during rearing. During pre‐breeding, we observed a negative correlation between rainfall and straightness of trajectories for males, but a positive correlation for females. The straightness of the trajectories of males during incubation and of males and females during rearing was also positively correlated with rainfall.

Daily mean temperature showed contrasting relationships with the proportion of time in flight, depending on phase and sex. The proportion of time in flight was positively correlated with temperature for males and females during incubation and for females during rearing, but negatively correlated for males during rearing and more marginally during pre‐breeding (−0.02 [−0.04, 0.00], *p* = .06). We observed that temperatures during rearing were positively correlated with the range of movement for females, but not for males. No other correlation was found between temperature and range of movement for other phases. Temperatures were also positively correlated with the straightness of the trajectory of males and females during pre‐breeding and for females during rearing, and marginally for males during rearing (0.02 [−0.00, 0.04], *p* = .07).

Regardless of the phase and for both sexes, the proportion of time in flight was higher when the wind speed was between 2.5 and 7.5 m s^−1^ (Appendix S5); the maximum proportion of time in flight observed was at a wind speed of around 5 m s^−1^. Below 2.5 m s^−1^ and above 7.5 m s^−1^ the proportion of time in flight decreased. The range of movement for males and females during pre‐breeding and for males during incubation and rearing was negatively correlated with wind speeds below 2.5 m s^−1^ and above 7.5 m s^−1^, with the maximum range of movement observed at a wind speed of around 5 m s^−1^. The range of movement of females during rearing was marginally positively correlated with wind speed (0.20 [−0.03, 0.43], *p* = .09). The straightness of trajectories of males during breeding and females during incubation was positively correlated with wind speeds below 2.5 m s^−1^ or above 7.5 m s^−1^, and the straightness of trajectories of females during pre‐breeding was negatively correlated with wind speed.

### 3.3 Relationship between movement behaviours and productivity

We found a negative relationship between the number of fledglings and the mean daily proportion of time in flight of males during the rearing (−0.26 [−0.51, −0.01], *p* = .03), and marginally during the incubation (−0.29 [−0.62, 0.02], *p* = .07). We did not find any significant relationship for females or for the two other movement behaviours.

## 4 DISCUSSION

**FIGURE 4:**
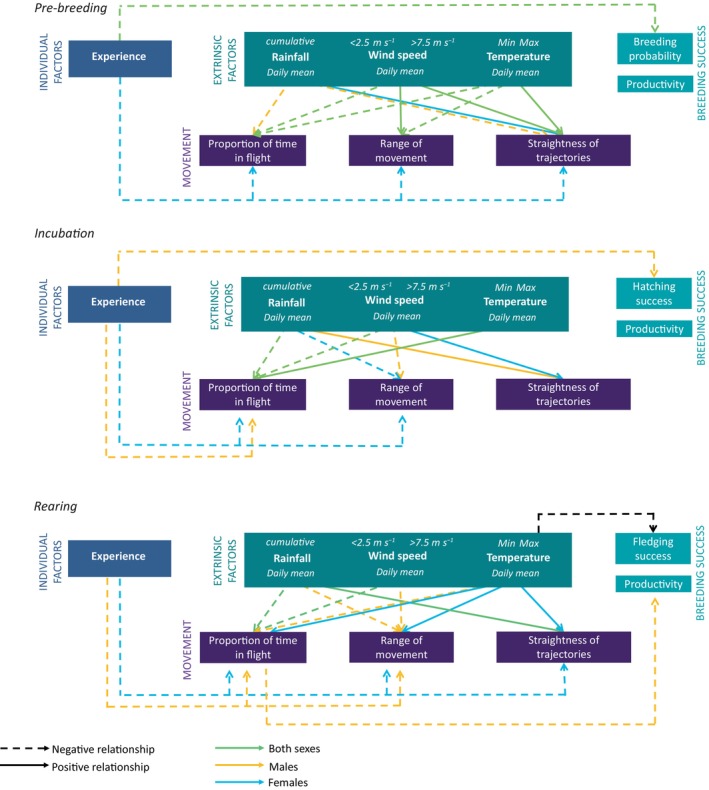
Relationships between the breeding performance of Bonelli's eagles, their movements (proportion of time in flight, range of movement and straightness of trajectories), intrinsic factors (experience, i.e. years spent on a breeding site for movement models and recruitment of the female, male or both on a breeding site for the breeding models), and local weather conditions (rainfall, wind speed and temperature). Only significant results are represented here. Solid arrows represent positive relationships and dotted arrows negative relationships (for the quadratic effect of wind speed, it is considered negative when the slope coefficient >0 and positive when it is <0). Blue, orange, green and black arrows respectively show relationships considering females, males, both sexes or none.

The results of our analyses indicate that variation in the breeding performance of the Bonelli's eagle in France was mainly driven by whether a new individual recruited into a territory (Figure 4). From GPS‐tracking data, we found large between‐individual variation in the three studied movement behaviours (proportion of time in flight, range of movement and sinuosity/straightness) at a daily scale and related to weather conditions (wind and rainfall). Contrary to our expectations, however, we found little evidence for a link between bird movement and breeding performance. We only detected that male Bonelli's eagles spending more time flying during the chick‐rearing phase had lower productivity. However, movement behaviours and breeding performance were indirectly linked through an individual's experience, with inexperienced birds spending more time flying, having a larger range of movement with straighter trajectories and lower breeding performance compared to experienced birds. These findings suggest that by adapting their daily movements and foraging strategies, Bonelli's eagles might be able to buffer the effect of adverse environmental conditions such as weather and prey availability on their breeding performance.

### 4.1 Local weather conditions strongly affect movements, but not breeding performance

We found that daily local weather conditions strongly impacted movement behaviours of individuals. Rainfall and wind speed constrained movements of Bonelli's eagles, especially the time they spend in flight. In rainy conditions, individuals spent less time flying, had a smaller range of movement and flew in straighter trajectories. It has been demonstrated that rain can negatively influence bird flight (Pastorino et al., 2017), compromising visibility and navigation (Newton, 2007). Soaked plumage increases wing loading and the energy costs associated with flying (Mahoney, 1984). Moreover, potential prey for eagles typically take cover during rainy weather, making them less detectable for the predator (Lehikoinen et al., 2009).

We also found that movements were affected by wind speed, with optimum movement between 2.5 and 7.5 m s^−1^. When wind speed is outside this optimum, individuals spent less time in flight, had a smaller range of movement and opted for straighter trajectories. Independently of landscape features (e.g. topography), both low and high wind speeds may force individuals to rely more on costly flapping flights (Amélineau et al., 2014; Furness & Bryant, 1996; Pennycuick, 1989). Several studies have shown that the flight capacity of birds is constrained by weather conditions, notably in birds relying on soaring flight (Duerr et al., 2015; Katzner et al., 2012; Shamoun‐Baranes et al., 2003, 2006). In turn, unfavourable weather conditions have been demonstrated to reduce foraging performance (Dawson & Bortolotti, 2000; McDonald et al., 2004; Newton & Marquiss, 1986; Penteriani, 1997; Sergio, 2003; Steenhof et al., 1997). For instance, rainfall during egg‐laying and the first week after hatching negatively impacted the breeding performance of numerous raptor species, such as the common buzzard (*Buteo Buteo*), northern goshawk (*Accipiter gentilis*) (Kostrzewa & Kostrzewa, 1990) and peregrine falcon (*Falco peregrinus*) (Anctil et al., 2014). We thus expected an effect of weather conditions on breeding performance through the alteration of individual movements. Others studies on other populations showed negative effect of precipitations on Bonelli's eagles breeding performances (López‐Peinado & López‐López, 2023). Yet, our results did not show that the breeding performance of Bonelli's eagles was affected by rain or wind, whatever the parameter considered (breeding probability, hatching or fledging success). It appears that individuals can modify their movements in response to weather conditions by adjusting their commuting and foraging strategies (Shamoun‐Baranes et al., 2016) as has been shown in the lesser kestrel (*Falco naumanni*) (Hernández‐Pliego et al., 2017). By adapting movements to weather conditions, Bonelli's eagles might be able to provide food to their chicks whatever the environmental conditions, ensuring breeding success. When weather conditions are bad, individuals might limit their movements by using more perching hunting techniques, which are less energy‐demanding than searching for prey while flying (Cecere et al., 2020). An alternative but not mutually exclusive hypothesis is that weather conditions at the scale of the breeding season might include sufficient periods with good conditions – for example, few days with rain (which is the case in the Mediterranean region) – to allow individuals to deal with adverse days.

Numerous studies have shown either a positive link between temperature and breeding performance of raptors (Kostrzewa & Kostrzewa, 1990; Lehikoinen et al., 2013) or a negative link (Steenhof et al., 1997; Tomback & Murphy, 1981). As the French population of Bonelli's eagles reaches the northern limit of the species' distribution, we did not expect a negative effect of maximum temperature on breeding performance. However, the results show that in fact in our study area breeding performance was strongly affected by higher maximum temperatures during rearing, whereas there was no effect of minimum temperature. Overall, temperature had a weaker effect on movement compared to rainfall and wind speed for both males and females. Temperature can influence food abundance by affecting lower trophic levels (Møller et al., 2010). It can also entail thermoregulation costs (Stevenson & Bryant, 2000). The negative effects of high temperature during rearing we observed could be the result of direct mortality of chicks. During the first days after hatching, nestlings do not thermoregulate, and their survival could highly depend on weather conditions (Elkins, 2010; Tomback & Murphy, 1981). During rearing, when temperatures were higher, females spent more time in flight, had a larger range of movement and flew in straighter trajectories. The combined effect of high temperatures on fledging success and the female's movements during rearing may indicate a shortage of prey, forcing the female to forage more or earlier in the season and negatively affecting chick survival. Outside the rearing phase for females, we did not find an effect of temperature on Bonelli's eagle movements, as shown in another raptor, the black kite (*Milvus migrans*), for which higher temperatures allow individuals to forage mainly by using soaring/gliding sequences (Sergio, 2003). After the chicks have fledged, we can hypothesise that the range of movement of individuals may be more flexible, as individuals do not need to stay close to the nest, allowing individuals to take advantage of favourable temperature conditions for soaring and thus have a greater range of movement.

### 4.2 Movements during the breeding season show sex‐specific differences

We observed differences in movement behaviours between sexes that depend on the phase of the breeding season, similar to what has already been observed in this species (López‐López et al., 2022; Martínez et al., 2020). Differences in movements depending on sex have been observed in other bird species (Lewis et al., 2002), including other raptors such as the golden eagle (*Aquila chrysaetos*) (Sur et al., 2020). Females and males might not adopt the same movement strategies (Clay et al., 2020). During incubation, Bonelli's eagle females mostly stay at the nest to incubate the eggs and move considerably less (less time in flight and a shorter range of movement) than males, which forage for the pair (Byholm et al., 2011; Eldegard & Sonerud, 2012; Martínez et al., 2020). During pre‐breeding and rearing, while both sexes show similar movements, they have different responses to intrinsic and extrinsic factors, which may be the result of sex‐specific pressures demanded by breeding. During incubation and early rearing, males are responsible for feeding the female and young and have strong pressure to forage (López‐López et al., 2022). This might make them less able to adapt their movements to local weather conditions, as is suggested by the smaller effect of wind speed that we observed on the time in flight and trajectory straightness of males during incubation and rearing, compared to the pre‐breeding phase and females. Beyond the differences associated with parental roles, we found that movement responses to extrinsic factors during breeding were also partly sex‐specific. For example, females' movements were more strongly affected by temperature during rearing, and wind conditions in all phases, compared to males. Like many raptors, female Bonelli's eagles are larger than males (Ferguson‐Lees & Christie, 2001), and might thus face higher energy costs related to movements (Brown, 1963; López‐López et al., 2022; Pennycuick, 2008). Females are probably more dependent on soaring flight and, consequently, on weather conditions favouring thermal uplifts (high daily temperature gradient and intermediate wind speed, Hernández‐Pliego et al., 2017; Klaassen et al., 2010; Williams et al., 2020). Males might be able to do more flapping flights, and so may be less limited by weather conditions.

### 4.3 Proportion of time in flight of males during rearing affects productivity

We expected that individuals with a higher proportion of time in flight, a larger range of movement and straighter trajectories, especially during the rearing period, would have reduced productivity, reflecting poorer‐quality breeding sites and/or individuals and a higher energy expenditure as a consequence (Nathan et al., 2008; Pennycuick, 1989). We did not find any effect of the straightness of trajectories or of the range of movement on productivity, whatever the sex and the phase, nor did we find any effect of the proportion of time in flight by females whatever the phase. The lack of effect of movement behaviours that we observed on the breeding performance of Bonelli's eagles suggest that flying may be less costly than we hypothesised. As many large birds, Bonelli's eagles mainly use soaring flight, which is considerably less costly than flapping flight (Duriez et al., 2014; Hedenström & Bone, 1997; Pennycuick, 2008), but rely on weather conditions favouriting thermal uplifts and/or topography favouring orographic uplifts. Thus, individuals might be able to perform long flights without much energy expenditure. However, males that spent more time in flight during rearing, fledged less chicks. As well, males with a higher proportion of time in flight during incubation tended to have lower productivity. A higher proportion of time in flight during incubation and rearing could reflect difficulties for the eagles to find enough prey to feed their chicks. Thus, it is therefore likely that males that spend a higher proportion of time in flight during incubation and rearing occupy poorer‐quality breeding sites with a lower prey availability, and/or are low‐quality individuals, requiring an increase in foraging effort. In our study, we have not considered the characteristics of territories and how they can impact movements and breeding performances (Newton, 1998). However, previous studies have observed little effect of breeding site quality on Bonelli's eagle breeding performance (Carrete et al., 2006; López‐López et al., 2007). Such results may come from our inability to reliably measure the quality of a territory for a generalist predator or may suggest that the variation in individual quality is actually larger than the variation in territory quality. These variations in the proportion of time in flight may also be related to interannual variation in weather conditions or prey availability.

### 4.4 Individual experience improves both movement efficiency and breeding performance

Apart from sexual dimorphism in breeding behaviour, the high diversity of individuals in terms of experience (1–15 years) in the study population might explain some of the observed heterogeneity in movements (Hertel et al., 2020; Patrick & Weimerskirch, 2014). Our results show that an individual's experience on its breeding site had a major impact both on its movements and its breeding performance. More experienced Bonelli's eagles seem to have more efficient movements, spending less time in flight, closer to the nest, with more sinuous trajectories.

Experienced birds tend to have higher breeding performance (Clutton‐Brock, 1988; Daunt et al., 2007; Pärt, 1997). The effect of age on breeding performance is well known in Bonelli's eagles, with young individuals having lower fecundity than older ones (Carrete et al., 2006; López‐Peinado & López‐López, 2023; Penteriani et al., 2003). We found that the breeding performance of Bonelli's eagles, notably breeding probability and hatching success, was severely reduced when one individual newly recruited, especially the female. The breeding probability was particularly low (0.46) when both male and female were new recruits. This negative effect of recruitment on breeding performance might be the consequence of a lack of shared experience between partners (Sánchez‐Macouzet et al., 2014; van de Pol et al., 2006; Wiley & Ridley, 2018), poor knowledge of foraging areas (Healy & Hurly, 2004), reduced hunting skills (Rutz et al., 2006), or the energy cost of the dispersal process and the settlement of a new territory (Bonte et al., 2012). A number of studies have shown the importance of memory and how individuals learn about their environment on the movement and habitat use of individuals (Avgar et al., 2013; Dalziel et al., 2008; Fagan et al., 2013; McClintock et al., 2012; Merkle et al., 2014). Thus, the positive effects of experience that we observed on breeding performance could also be linked to the effect of experience on movement efficiency, highlighting a possible indirect link between movements and breeding performance. As individuals acquire information about their environment (Fagan et al., 2013) and improve their navigating and orientation skills (Mueller et al., 2013; Thorup et al., 2007) as well as their hunting efficiency (Rutz et al., 2006), they learn how to optimise their movements and the associated energy costs (Maransky & Bildstein, 2001), improving their breeding performance.

## 5 CONCLUSION

The relationships between movements, breeding performance and experience that we found in the Bonelli's eagle support the hypothesis that experienced individuals gradually acquire knowledge about their territory allowing them to develop adequate foraging strategies in response to e.g. weather conditions (Grubb, 1975; Krebs & Davies, 2009; Miller et al., 2016; Redpath et al., 2002). Experience may also play an important role in habitat use (Fagan et al., 2013), and it would be interesting to investigate if experience affects how Bonelli's eagles use different habitats available in their territory and how this may affect breeding success. Finally, and for a comparative purpose, it would be relevant to carry out similar studies on other bird species with different life histories (from short‐lived to long‐lived) and flight modes (from active flapping flight to obligate soaring).

## AUTHOR CONTRIBUTIONS

**Lise Viollat:** Conceptualization (equal); data curation (equal); formal analysis (equal); methodology (equal); visualization (equal); writing – original draft (equal). **Alexandre Millon:** Conceptualization (equal); methodology (equal); supervision (equal); writing – review and editing (equal). **Cécile Ponchon:** Funding acquisition (equal); investigation (equal); writing – review and editing (equal). **Alain Ravayrol:** Investigation (equal); writing – review and editing (equal). **Thibaut Couturier:** Methodology (equal); writing – review and editing (equal). **Aurélien Besnard:** Conceptualization (equal); methodology (equal); supervision (equal); writing – review and editing (equal).

## CONFLICT OF INTEREST STATEMENT

The authors declare no conflicts of interest.

## Supporting information


Appendix S1

Appendix S2

Appendix S3

Appendix S4

Appendix S5

Appendix S6

## Data Availability

The codes and data that support the findings of this study are available on Zenodo at: https://doi.org/10.5281/zenodo.11474146.

## References

[ece370081-bib-0001] Allen, A. M. , & Singh, N. J. (2016). Linking movement ecology with wildlife management and conservation. Frontiers in Ecology and Evolution, 3, 155.

[ece370081-bib-0002] Amélineau, F. , Péron, C. , Lescroel, A. , Authier, M. , Provost, P. , & Grémillet, D. (2014). Windscape and tortuosity shape the flight costs of northern gannets. The Journal of Experimental Biology, 217, 876–885. 10.1242/jeb.097915 24622894

[ece370081-bib-0003] Anctil, A. , Franke, A. , & Bêty, J. (2014). Heavy rainfall increases nestling mortality of an arctic top predator: Experimental evidence and long‐term trend in peregrine falcons. Oecologia, 174, 1033–1043. 10.1007/s00442-013-2800-y 24135996 PMC3933744

[ece370081-bib-0004] Avgar, T. , Deardon, R. , & Fryxell, J. M. (2013). An empirically parameterized individual based model of animal movement, perception, and memory. Ecological Modelling, 251, 158–172. 10.1016/j.ecolmodel.2012.12.002

[ece370081-bib-0005] Batschelet, E. (1981). Circular statistics in biology. Academic Press.

[ece370081-bib-0006] Bjørnstad, O. N. , & Hansen, T. F. (1994). Individual variation and population dynamics. Oikos, 69, 167–171. 10.2307/3545298

[ece370081-bib-0007] Bonte, D. , Van Dyck, H. , Bullock, J. M. , Coulon, A. , Delgado, M. , Gibbs, M. , Lehouck, V. , Matthysen, E. , Mustin, K. , Saastamoinen, M. , Schtickzelle, N. , Stevens, V. M. , Vandewoestijne, S. , Baguette, M. , Barton, K. , Benton, T. G. , Chaput‐Bardy, A. , Clobert, J. , Dytham, C. , … Travis, J. M. J. (2012). Costs of dispersal. Biological Reviews, 87, 290–312. 10.1111/j.1469-185X.2011.00201.x 21929715

[ece370081-bib-0008] Börger, L. , Dalziel, B. D. , & Fryxell, J. M. (2008). Are there general mechanisms of animal home range behaviour? A review and prospects for future research. Ecology Letters, 11, 637–650. 10.1111/j.1461-0248.2008.01182.x 18400017

[ece370081-bib-0009] Bradley, J. S. , Wooller, R. D. , & Skira, I. J. (2000). Intermittent breeding in the short‐tailed shearwater *Puffinus tenuirostris* . Journal of Animal Ecology, 69, 639–650. 10.1046/j.1365-2656.2000.00422.x

[ece370081-bib-0010] Brooks, M. E. , Kristensen, K. , van Benthem, K. J. , Magnusson, A. , Berg, C. W. , Nielsen, A. , Skaug, H. J. , Mächler, M. , & Bolker, B. M. (2017). glmmTMB balances speed and flexibility among packages for zero‐inflated generalized linear mixed modeling. The R Journal, 9, 378. 10.32614/RJ-2017-066

[ece370081-bib-0011] Brown, R. H. J. (1963). The flight of birds. Biological Reviews, 38, 460–489. 10.1111/j.1469-185X.1963.tb00790.x

[ece370081-bib-0012] Burger, J. , Hiessler, N. , Ponchon, C. , & Vincent‐Martin, N. (2013). Troisième plan national d'actions en faveur de l'Aigle de Bonelli 2014‐2023. Conservatoire d'espaces naturels du Languedoc‐Roussillon, Conservatoire d'espaces naturels de PACA et Ministère de l'écologie, du développement durable et de l'énergie.

[ece370081-bib-0013] Burt, W. H. (1943). Territoriality and home range concepts as applied to mammals. Journal of Mammalogy, 24, 346–352. 10.2307/1374834

[ece370081-bib-0014] Byholm, P. , Rousi, H. , & Sole, I. (2011). Parental care in nesting hawks: Breeding experience and food availability influence the outcome. Behavioral Ecology, 22, 609–615. 10.1093/beheco/arr019

[ece370081-bib-0015] Calenge, C. (2006). The package “adehabitat” for the R software: A tool for the analysis of space and habitat use by animals. Ecological Modelling, 197, 516–519. 10.1016/j.ecolmodel.2006.03.017

[ece370081-bib-0016] Cam, E. , Gimenez, O. , Alpizar‐Jara, R. , Aubry, L. M. , Authier, M. , Cooch, E. G. , Koons, D. N. , Link, W. A. , Monnat, J.‐Y. , Nichols, J. D. , Rotella, J. J. , Royle, J. A. , & Pradel, R. (2013). Looking for a needle in a haystack: Inference about individual fitness components in a heterogeneous population. Oikos, 122, 739–753. 10.1111/j.1600-0706.2012.20532.x

[ece370081-bib-0017] Carrete, M. , Sánchez‐Zapata, J. A. , Tella, J. L. , Gil‐Sánchez, J. M. , & Moleón, M. (2006). Components of breeding performance in two competing species: Habitat heterogeneity, individual quality and density‐dependence. Oikos, 112, 680–690. 10.1111/j.0030-1299.2006.14528.x

[ece370081-bib-0018] Cecere, J. G. , De Pascalis, F. , Imperio, S. , Ménard, D. , Catoni, C. , Griggio, M. , & Rubolini, D. (2020). Inter‐individual differences in foraging tactics of a colonial raptor: Consistency, weather effects, and fitness correlates. Movement Ecology, 8, 28. 10.1186/s40462-020-00206-w 32587702 PMC7313117

[ece370081-bib-0019] Chevallier, C. , Hernández‐Matías, A. , Real, J. , Vincent‐Martin, N. , Ravayrol, A. , & Besnard, A. (2015). Retrofitting of power lines effectively reduces mortality by electrocution in large birds: An example with the endangered Bonelli's eagle. Journal of Applied Ecology, 52, 1465–1473. 10.1111/1365-2664.12476

[ece370081-bib-0020] Clay, T. A. , Joo, R. , Weimerskirch, H. , Phillips, R. A. , den Ouden, O. , Basille, M. , Clusella‐Trullas, S. , Assink, J. D. , & Patrick, S. C. (2020). Sex‐specific effects of wind on the flight decisions of a sexually dimorphic soaring bird. Journal of Animal Ecology, 89, 1811–1823. 10.1111/1365-2656.13267 32557603

[ece370081-bib-0021] Clutton‐Brock, T. H. (1988). Reproductive success: Studies of individual variation in contrasting breeding systems. University of Chicago Press.

[ece370081-bib-0022] Cooke, S. J. , Hinch, S. G. , Wikelski, M. , Andrews, R. D. , Kuchel, L. J. , Wolcott, T. G. , & Butler, P. J. (2004). Biotelemetry: A mechanistic approach to ecology. Trends in Ecology & Evolution, 19, 334–343. 10.1016/j.tree.2004.04.003 16701280

[ece370081-bib-0023] Covas, R. , Doutrelant, C. , & Du Plessis, M. A. (2004). Experimental evidence of a link between breeding conditions and the decision to breed or to help in a colonial cooperative bird. Proceedings of the Royal Society of London. Series B: Biological Sciences, 271, 827–832. 10.1098/rspb.2003.2652 PMC1691664 15255101

[ece370081-bib-0024] Dalziel, B. , Morales, J. , & Fryxell, J. (2008). Fitting probability distributions to animal movement trajectories: Using artificial neural networks to link distance, resources, and memory. The American Naturalist, 172, 248–258. 10.1086/589448 18598199

[ece370081-bib-0025] Daunt, F. , Wanless, S. , Harris, M. P. , Money, L. , & Monaghan, P. (2007). Older and wiser: Improvements in breeding success are linked to better foraging performance in European shags. Functional Ecology, 21, 561–567. 10.1111/j.1365-2435.2007.01260.x

[ece370081-bib-0026] Dawson, R. D. , & Bortolotti, G. R. (2000). Reproductive success of American kestrels: The role of prey abundance and weather. The Condor, 102, 814–822. 10.1093/condor/102.4.814

[ece370081-bib-0027] Del Hoyo, J. , Elliot, A. , & Sargatal, J. (1994). Handbook of the birds of the world, vol 2. New world vultures to Guineafowl. Lynx ed.

[ece370081-bib-0028] Duerr, A. E. , Miller, T. A. , Lanzone, M. , Brandes, D. , Cooper, J. , O'Malley, K. , Maisonneuve, C. , Tremblay, J. A. , & Katzner, T. (2015). Flight response of slope‐soaring birds to seasonal variation in thermal generation. Functional Ecology, 29, 779–790. 10.1111/1365-2435.12381

[ece370081-bib-0029] Duriez, O. , Kato, A. , Tromp, C. , Dell'Omo, G. , Vyssotski, A. L. , Sarrazin, F. , & Ropert‐Coudert, Y. (2014). How cheap is soaring flight in raptors? A preliminary investigation in freely‐flying vultures. PLoS One, 9, e84887. 10.1371/journal.pone.0084887 24454760 PMC3893159

[ece370081-bib-0030] Dussault, C. , Pinard, V. , Ouellet, J.‐P. , Courtois, R. , & Fortin, D. (2012). Avoidance of roads and selection for recent cutovers by threatened caribou: Fitness‐rewarding or maladaptive behaviour? Proceedings of the Royal Society of London. Series B: Biological Sciences, 279, 4481–4488. 10.1098/rspb.2012.1700 PMC3479810 22951736

[ece370081-bib-0031] Eldegard, K. , & Sonerud, G. A. (2012). Sex roles during post‐fledging care in birds: Female Tengmalm's owls contribute little to food provisioning. Journal für Ornithologie, 153, 385–398. 10.1007/s10336-011-0753-7

[ece370081-bib-0032] Elkins, N. (2010). Weather and bird behaviour. Bloomsbury Publishing.

[ece370081-bib-0033] Fagan, W. F. , Lewis, M. A. , Auger‐Méthé, M. , Avgar, T. , Benhamou, S. , Breed, G. , LaDage, L. , Schlägel, U. E. , Tang, W. , Papastamatiou, Y. P. , Forester, J. , & Mueller, T. (2013). Spatial memory and animal movement. Ecology Letters, 16, 1316–1329. 10.1111/ele.12165 23953128

[ece370081-bib-0034] Ferguson‐Lees, J. , & Christie, D. A. (2001). Raptors of the world. Houghton Mifflin Harcourt.

[ece370081-bib-0035] Furness, R. W. , & Bryant, D. M. (1996). Effect of wind on field metabolic rates of breeding northern fulmars. Ecology, 77, 1181–1188. 10.2307/2265587

[ece370081-bib-0036] García, V. , Iglesias‐Lebrija, J. J. , & Moreno‐Opo, R. (2021). Null effects of the Garcelon harnessing method and transmitter type on soaring raptors. Ibis, 163, 899–912. 10.1111/ibi.12942

[ece370081-bib-0037] García, V. , Moreno‐Opo, R. , & Tintó, A. (2013). Sex differentiation of Bonelli's eagle *Aquila fasciata* in Western Europe using morphometrics and plumage colour patterns. Ardeola: Revista Ibérica de Ornitología, 60, 261–277. 10.13157/arla.60.2.2013.261

[ece370081-bib-0038] Gil‐Sánchez, J. M. , Moleón, M. , Otero, M. , & Bautista, J. (2004). A nine‐year study of successful breeding in a Bonelli's eagle population in southeast Spain: A basis for conservation. Biological Conservation, 118, 685–694. 10.1016/j.biocon.2003.10.017

[ece370081-bib-0039] Gimenez, O. , Cam, E. , & Gaillard, J.‐M. (2018). Individual heterogeneity and capture‐recapture models: What, why and how? Oikos, 127, 664–686. 10.1111/oik.04532

[ece370081-bib-0040] Grubb, T. C. (1975). Weather‐dependent foraging behavior of some birds wintering in a deciduous woodland. The Condor, 77, 175–182. 10.2307/1365788

[ece370081-bib-0041] Gupte, P. R. , Beardsworth, C. E. , Spiegel, O. , Lourie, E. , Toledo, S. , Nathan, R. , & Bijleveld, A. I. (2021). A guide to pre‐processing high‐throughput animal tracking data. Journal of Animal Ecology, 91, 287–307. 10.1111/1365-2656.13610 34657296 PMC9299236

[ece370081-bib-0042] Hamel, S. , Gaillard, J.‐M. , Yoccoz, N. G. , Loison, A. , Bonenfant, C. , & Descamps, S. (2010). Fitness costs of reproduction depend on life speed: Empirical evidence from mammalian populations. Ecology Letters, 13, 915–935. 10.1111/j.1461-0248.2010.01478.x 20482573

[ece370081-bib-0043] Healy, S. D. , & Hurly, T. A. (2004). Spatial learning and memory in birds. Brain, Behavior and Evolution, 63, 211–220. 10.1159/000076782 15084814

[ece370081-bib-0044] Hedenström, A. , & Bone, Q. (1997). Migration by soaring or flapping flight in birds: The relative importance of energy cost and speed. Philosophical Transactions of the Royal Society of London. Series B: Biological Sciences, 342, 353–361. 10.1098/rstb.1993.0164

[ece370081-bib-0045] Hernández‐Matías, A. , Real, J. , Moleón, M. , Palma, L. , Sánchez‐Zapata, J. A. , Pradel, R. , Carrete, M. , Gil‐Sánchez, J. M. , Beja, P. , Balbontín, J. , Vincent‐Martin, N. , Ravayrol, A. , Benítez, J. R. , Arroyo, B. , Fernández, C. , Ferreiro, E. , & García, J. (2013). From local monitoring to a broad‐scale viability assessment: A case study for the Bonelli's eagle in western Europe. Ecological Monographs, 83, 239–261. 10.1890/12-1248.1

[ece370081-bib-0046] Hernández‐Matías, A. , Real, J. , Parés, F. , & Pradel, R. (2015). Electrocution threatens the viability of populations of the endangered Bonelli's eagle (*Aquila fasciata*) in southern Europe. Biological Conservation, 191, 110–116. 10.1016/j.biocon.2015.06.028

[ece370081-bib-0047] Hernández‐Matías, A. , Real, J. , Pradel, R. , Ravayrol, A. , & Vincent‐Martin, N. (2011). Effects of age, territoriality and breeding on survival of Bonelli's eagle *Aquila fasciata* . Ibis, 153, 846–857. 10.1111/j.1474-919X.2011.01158.x

[ece370081-bib-0048] Hernández‐Matías, A. , Real, J. , Pradel, R. , Ravayrol, A. , Vincent‐Martin, N. , Bosca, F. , & Cheylan, G. (2010). Determinants of territorial recruitment in Bonelli's eagle (*Aquila fasciata*) populations. The Auk, 127, 173–184. 10.1525/auk.2009.09143

[ece370081-bib-0049] Hernández‐Pliego, J. , Rodríguez, C. , Dell'Omo, G. , & Bustamante, J. (2017). Combined use of tri‐axial accelerometers and GPS reveals the flexible foraging strategy of a bird in relation to weather conditions. PLoS One, 12, e0177892. 10.1371/journal.pone.0177892 28591181 PMC5462363

[ece370081-bib-0050] Hertel, A. G. , Niemelä, P. T. , Dingemanse, N. J. , & Mueller, T. (2020). A guide for studying among‐individual behavioral variation from movement data in the wild. Movement Ecology, 8, 30. 10.1186/s40462-020-00216-8 32612837 PMC7325061

[ece370081-bib-0051] Hijmans, R. J. , & Karney, C. (GeographicLib), (2022). *Spherical trigonometry for geographic applications* In Williams and Chris Vennes (Eds.), Geosphere: Spherical trigonometry. Available at: https://cran.r‐project.org/web/packages/geosphere/index.html

[ece370081-bib-0052] Jenouvrier, S. , Péron, C. , & Weimerskirch, H. (2015). Extreme climate events and individual heterogeneity shape life‐history traits and population dynamics. Ecological Monographs, 85, 605–624. 10.1890/14-1834.1

[ece370081-bib-0053] Katzner, T. E. , Brandes, D. , Miller, T. , Lanzone, M. , Maisonneuve, C. , Tremblay, J. A. , Mulvihill, R. , & Merovich, G. T., Jr. (2012). Topography drives migratory flight altitude of golden eagles: Implications for on‐shore wind energy development. Journal of Applied Ecology, 49, 1178–1186. 10.1111/j.1365-2664.2012.02185.x

[ece370081-bib-0054] Kendall, B. E. , Fox, G. A. , Fujiwara, M. , & Nogeire, T. M. (2011). Demographic heterogeneity, cohort selection, and population growth. Ecology, 92, 1985–1993. 10.1890/11-0079.1 22073789

[ece370081-bib-0055] Klaassen, R. H. G. , Hake, M. , Strandberg, R. , & Alerstam, T. (2010). Geographical and temporal flexibility in the response to crosswinds by migrating raptors. Proceedings of the Royal Society of London. Series B: Biological Sciences, 278, 1339–1346. 10.1098/rspb.2010.2106 PMC3061148 20980299

[ece370081-bib-0057] Kostrzewa, A. , & Kostrzewa, R. (1990). The relationship of spring and summer weather with density and breeding performance of the Buzzard *Buteo buteo*, Goshawk *Accipiter gentilis* and Kestrel *Falco tinnunculus* . Ibis, 132, 550–559. 10.1111/j.1474-919X.1990.tb00278.x

[ece370081-bib-0058] Krebs, J. R. , & Davies, N. B. (2009). Behavioural ecology: An evolutionary approach. John Wiley & Sons.

[ece370081-bib-0059] Lande, R. , Engen, S. , & Saether, B.‐E. (2003). Stochastic population dynamics in ecology and conservation. Oxford University Press. 10.1093/acprof:oso/9780198525257.001.0001

[ece370081-bib-0060] Lehikoinen, A. , Byholm, P. , Ranta, E. , Saurola, P. , Valkama, J. , Korpimäki, E. , Pietiäinen, H. , & Henttonen, H. (2009). Reproduction of the common buzzard at its northern range margin under climatic change. Oikos, 118, 829–836. 10.1111/j.1600-0706.2008.17440.x

[ece370081-bib-0061] Lehikoinen, A. , Lindén, A. , Byholm, P. , Ranta, E. , Saurola, P. , Valkama, J. , Kaitala, V. , & Lindén, H. (2013). Impact of climate change and prey abundance on nesting success of a top predator, the goshawk. Oecologia, 171, 283–293. 10.1007/s00442-012-2411-z 22791186

[ece370081-bib-0062] Lewis, S. , Benvenuti, S. , Dall'Antonia, L. , Griffiths, R. , Money, L. , Sherratt, T. N. , Wanless, S. , & Hamer, K. C. (2002). Sex‐specific foraging behaviour in a monomorphic seabird. Proceedings of the Royal Society of London. Series B: Biological Sciences, 269, 1687–1693. 10.1098/rspb.2002.2083 PMC1691079 12204129

[ece370081-bib-0063] Lieury, N. , Besnard, A. , Ponchon, C. , Ravayrol, A. , & Millon, A. (2016). Geographically isolated but demographically connected: Immigration supports efficient conservation actions in the recovery of a range‐margin population of the Bonelli's eagle in France. Biological Conservation, 195, 272–278. 10.1016/j.biocon.2016.01.011

[ece370081-bib-0064] Lomnicki, A. (1978). Individual differences between animals and the natural regulation of their numbers. The Journal of Animal Ecology, 47, 461. 10.2307/3794

[ece370081-bib-0065] López‐López, P. , García‐Ripollés, C. , & Urios, V. (2007). Population size, breeding performance and territory quality of Bonelli's eagle *Hieraaetus fasciatus* in eastern Spain. Bird Study, 54, 335–342. 10.1080/00063650709461493

[ece370081-bib-0066] López‐López, P. , Perona, A. M. , Egea‐Casas, O. , Morant, J. , & Urios, V. (2022). Tri‐axial accelerometry shows differences in energy expenditure and parental effort throughout the breeding season in long‐lived raptors. Current Zoology, 68, 57–67. 10.1093/cz/zoab010 35169629 PMC8836325

[ece370081-bib-0067] López‐Peinado, A. , & López‐López, P. (2023). Breeders' age, nest‐site characteristics and climatic conditions but not density‐dependent effects determine Bonelli's eagle breeding performance: A long‐term study (2002–2021). Ornithological Applications, 126, duad048. 10.1093/ornithapp/duad048

[ece370081-bib-0068] Mahoney, S. A. (1984). Plumage wettability of aquatic birds. The Auk, 101, 181–185.

[ece370081-bib-0069] Maransky, B. P. , & Bildstein, K. L. (2001). Follow your elders: Age‐related differences in the migration behavior of broad‐winged hawks at Hawk Mountain Sanctuary, Pennsylvania. The Wilson Bulletin, 113, 350–353. 10.1676/0043-5643(2001)113[0350:FYEARD]2.0.CO;2

[ece370081-bib-0070] Martínez, J. E. , Zuberogoitia, I. , Escarabajal, J. M. , Cerezo, E. , Calvo, J. F. , & Margalida, A. (2020). Breeding behaviour and time‐activity budgets of Bonelli's eagles *Aquila fasciata*: Marked sexual differences in parental activities. Bird Study, 67, 35–44. 10.1080/00063657.2020.1733487

[ece370081-bib-0071] McClintock, B. T. , King, R. , Thomas, L. , Matthiopoulos, J. , McConnell, B. J. , & Morales, J. M. (2012). A general discrete‐time modeling framework for animal movement using multistate random walks. Ecological Monographs, 82, 335–349. 10.1890/11-0326.1

[ece370081-bib-0072] McDonald, P. G. , Olsen, P. D. , & Cockburn, A. (2004). Weather dictates reproductive success and survival in the Australian brown falcon *Falco berigora* . Journal of Animal Ecology, 73, 683–692. 10.1111/j.0021-8790.2004.00842.x

[ece370081-bib-0073] McDonald, T. L. , & White, G. C. (2010). A comparison of regression models for small counts. The Journal of Wildlife Management, 74, 514–521. 10.2193/2009-270

[ece370081-bib-0074] McLean, D. J. , & Skowron Volponi, M. A. (2018). trajr: An R package for characterisation of animal trajectories. Ethology, 124, 440–448. 10.1111/eth.12739

[ece370081-bib-0075] McLoughlin, P. D. , Boyce, M. S. , Coulson, T. , & Clutton‐Brock, T. (2006). Lifetime reproductive success and density‐dependent, multi‐variable resource selection. Proceedings of the Royal Society of London. Series B: Biological Sciences, 273, 1449–1454. 10.1098/rspb.2006.3486 PMC1560319 16777736

[ece370081-bib-0076] Merkle, J. A. , Fortin, D. , & Morales, J. M. (2014). A memory‐based foraging tactic reveals an adaptive mechanism for restricted space use. Ecology Letters, 17, 924–931. 10.1111/ele.12294 24811575

[ece370081-bib-0077] Miller, T. A. , Brooks, R. P. , Lanzone, M. J. , Brandes, D. , Cooper, J. , Tremblay, J. A. , Wilhelm, J. , Duerr, A. , & Katzner, T. E. (2016). Limitations and mechanisms influencing the migratory performance of soaring birds. Ibis, 158, 116–134. 10.1111/ibi.12331

[ece370081-bib-0078] Møller, A. P. , Fiedler, W. , & Berthold, P. (2010). Effects of climate change on birds. OUP.

[ece370081-bib-0079] Morales, J. M. , Moorcroft, P. R. , Matthiopoulos, J. , Frair, J. L. , Kie, J. G. , Powell, R. A. , Merrill, E. H. , & Haydon, D. T. (2010). Building the bridge between animal movement and population dynamics. Philosophical Transactions of the Royal Society of London. Series B, Biological Sciences, 365, 2289–2301. 10.1098/rstb.2010.0082 20566505 PMC2894961

[ece370081-bib-0080] Mourocq, E. , Bize, P. , Bouwhuis, S. , Bradley, R. , Charmantier, A. , de la Cruz, C. , Drobniak, S. M. , Espie, R. H. M. , Herényi, M. , Hötker, H. , Krüger, O. , Marzluff, J. , Møller, A. P. , Nakagawa, S. , Phillips, R. A. , Radford, A. N. , Roulin, A. , Török, J. , Valencia, J. , … Griesser, M. (2016). Life span and reproductive cost explain interspecific variation in the optimal onset of reproduction. Evolution, 70, 296–313. 10.1111/evo.12853 26763090

[ece370081-bib-0081] Mueller, T. , & Fagan, W. F. (2008). Search and navigation in dynamic environments – From individual behaviors to population distributions. Oikos, 117, 654–664.

[ece370081-bib-0082] Mueller, T. , O'Hara, R. B. , Converse, S. J. , Urbanek, R. P. , & Fagan, W. F. (2013). Social learning of migratory performance. Science, 341, 999–1002. 10.1126/science.1237139 23990559

[ece370081-bib-0083] Nathan, R. , Getz, W. M. , Revilla, E. , Holyoak, M. , Kadmon, R. , Saltz, D. , & Smouse, P. E. (2008). A movement ecology paradigm for unifying organismal movement research. Proceedings of the National Academy of Sciences of the United States of America, 105, 19052–19059. 10.1073/pnas.0800375105 19060196 PMC2614714

[ece370081-bib-0084] Newton, I. (1989). Lifetime reproduction in birds. Academic Press.

[ece370081-bib-0085] Newton, I. (1998). Population limitation in birds. Academic Press.

[ece370081-bib-0086] Newton, I. (2007). Weather‐related mass‐mortality events in migrants. Ibis, 149, 453–467. 10.1111/j.1474-919X.2007.00704.x

[ece370081-bib-0087] Newton, I. , & Marquiss, M. (1986). Population regulation in sparrowhawks. Journal of Animal Ecology, 55, 463–480. 10.2307/4731

[ece370081-bib-0088] Pärt, T. (1997). Does breeding experience explain increased reproductive success with age? An experiment. Proceedings of the Royal Society of London. Series B: Biological Sciences, 260, 113–117. 10.1098/rspb.1995.0067

[ece370081-bib-0089] Pastorino, A. , Roman, J. R. , Agostini, N. , Dell'Omo, G. , & Panuccio, M. (2017). Fog and rain lead migrating White storks *Ciconia ciconia* to perform reverse migration and to land. Avocetta, 41, 5–12.

[ece370081-bib-0090] Patrick, S. C. , & Weimerskirch, H. (2014). Consistency pays: Sex differences and fitness consequences of behavioural specialization in a wide‐ranging seabird. Biology Letters, 10, 20140630. 10.1098/rsbl.2014.0630 25354918 PMC4272207

[ece370081-bib-0091] Pennycuick, C. (1989). Bird flight performance: A practical calculation manual. Oxford University Press.

[ece370081-bib-0092] Pennycuick, C. J. (2008). Modelling the flying bird. Elsevier.

[ece370081-bib-0093] Penteriani, V. (1997). Long‐term study of a goshawk breeding population on a Mediterranean mountain (*Abruzzi apennines*, central Italy): Density, breeding performance and diet. Journal of Raptor Research, 31, 308–312.

[ece370081-bib-0094] Penteriani, V. , Balbontin, J. , & Ferrer, M. (2003). Simultaneous effects of age and territory quality on fecundity in Bonelli's eagle *Hieraaetus fasciatus* . Ibis, 145, E77–E82. 10.1046/j.1474-919X.2003.00159.x

[ece370081-bib-0095] Penteriani, V. , & Delgado, M. M. (2009). Thoughts on natal dispersal. Journal of Raptor Research, 43, 90–98. 10.3356/JRR-08-39.1

[ece370081-bib-0096] Pyke, G. (2019). Animal movements: An optimal foraging approach. In J. C. Choe (Ed.), Encyclopedia of animal behavior (pp. 149–156). Elsevier Academic Press. 10.1016/B978-0-12-809633-8.90160-2

[ece370081-bib-0097] Real, J. , Mañosa, S. , & Codina, J. (1998). Post‐nestling dependence period in the Bonelli's eagle *Hieraaetus fasciatus* . Ornis Fennica, 75, 129–137.

[ece370081-bib-0098] Redpath, S. M. , Arroyo, B. E. , Etheridge, B. , Leckie, F. , Bouwman, K. , & Thirgood, S. J. (2002). Temperature and hen harrier productivity: From local mechanisms to geographical patterns. Ecography, 25, 533–540. 10.1034/j.1600-0587.2002.250503.x

[ece370081-bib-0099] Rutz, C. , & Hays, G. C. (2009). New frontiers in biologging science. Biology Letters, 5, 289–292. 10.1098/rsbl.2009.0089 19324624 PMC2679933

[ece370081-bib-0100] Rutz, C. , Whittingham, M. J. , & Newton, I. (2006). Age‐dependent diet choice in an avian top predator. Proceedings of the Royal Society of London. Series B: Biological Sciences, 273, 579–586. 10.1098/rspb.2005.3353 PMC1560053 16537129

[ece370081-bib-0101] Sánchez‐Macouzet, O. , Rodríguez, C. , & Drummond, H. (2014). Better stay together: Pair bond duration increases individual fitness independent of age‐related variation. Proceedings of the Royal Society of London. Series B: Biological Sciences, 281, 20132843. 10.1098/rspb.2013.2843 PMC4046394 24827435

[ece370081-bib-0102] Seegar, W. S. , Cutchis, P. N. , Fuller, M. R. , Suter, J. J. , Bhatnagar, V. , & Wall, J. G. (1996). Fifteen years of satellite tracking development and application to wildlife research and conservation. Johns Hopkins APL Technical Digest, 17(4), 401–411.

[ece370081-bib-0103] Sergio, F. (2003). From individual behaviour to population pattern: Weather‐dependent foraging and breeding performance in black kites. Animal Behaviour, 66, 1109–1117. 10.1006/anbe.2003.2303

[ece370081-bib-0104] Shamoun‐Baranes, J. , Bouten, W. , van Loon, E. E. , Meijer, C. , & Camphuysen, C. J. (2016). Flap or soar? How a flight generalist responds to its aerial environment. Philosophical Transactions of the Royal Society, B: Biological Sciences, 371, 20150395. 10.1098/rstb.2015.0395 PMC4992719 27528785

[ece370081-bib-0105] Shamoun‐Baranes, J. , Liechti, O. , Yom‐Tov, Y. , & Leshem, Y. (2003). Using a convection model to predict altitudes of white stork migration over Central Israel. Boundary‐Layer Meteorology, 107, 673–681. 10.1023/A:1022824008388

[ece370081-bib-0106] Shamoun‐Baranes, J. , Van Loon, E. , Alon, D. , Alpert, P. , Yom‐Tov, Y. , & Leshem, Y. (2006). Is there a connection between weather at departure sites, onset of migration and timing of soaring‐bird autumn migration in Israel? Global Ecology and Biogeography, 15, 541–552. 10.1111/j.1466-8238.2006.00261.x

[ece370081-bib-0107] Shaw, A. K. , & Levin, S. A. (2013). The evolution of intermittent breeding. Journal of Mathematical Biology, 66, 685–703. 10.1007/s00285-012-0603-0 23076830

[ece370081-bib-0108] Shepard, E. L. C. , & Lambertucci, S. A. (2013). From daily movements to population distributions: Weather affects competitive ability in a guild of soaring birds. Journal of the Royal Society Interface, 10, 20130612. 10.1098/rsif.2013.0612 24026471 PMC3785828

[ece370081-bib-0109] Silva, R. , Afán, I. , Gil, J. A. , & Bustamante, J. (2017). Seasonal and circadian biases in bird tracking with solar GPS‐tags. PLoS One, 12, e0185344. 10.1371/journal.pone.0185344 29020062 PMC5636103

[ece370081-bib-0110] Stearns, S. C. (1992). The evolution of life histories. Oxford University Press. 10.1093/oso/9780198577416.001.0001

[ece370081-bib-0111] Steenhof, K. , Kochert, M. N. , & Mcdonald, T. L. (1997). Interactive effects of prey and weather on Golden eagle reproduction. The Journal of Animal Ecology, 66, 350–362. 10.2307/5981

[ece370081-bib-0112] Stevenson, I. R. , & Bryant, D. M. (2000). Climate change and constraints on breeding. Nature, 406, 366–367. 10.1038/35019151 10935624

[ece370081-bib-0113] Sur, M. , Duerr, A. E. , Bell, D. A. , Fisher, R. N. , Tracey, J. A. , Bloom, P. H. , Miller, T. A. , & Katzner, T. E. (2020). Relevance of individual and environmental drivers of movement of Golden eagles. Ibis, 162, 381–399. 10.1111/ibi.12766

[ece370081-bib-0114] Thorup, K. , Bisson, I.‐A. , Bowlin, M. S. , Holland, R. A. , Wingfield, J. C. , Ramenofsky, M. , & Wikelski, M. (2007). Evidence for a navigational map stretching across the continental U.S. in a migratory songbird. Proceedings of the National Academy of Sciences of the United States of America, 104, 18115–18119. 10.1073/pnas.0704734104 17986618 PMC2084305

[ece370081-bib-0115] Tomback, D. F. , & Murphy, J. R. (1981). Food deprivation and temperature regulation in nestling ferruginous hawks. The Wilson Bulletin, 93, 92–97.

[ece370081-bib-0116] van de Pol, M. , Heg, D. , Bruinzeel, L. W. , Kuijper, B. , & Verhulst, S. (2006). Experimental evidence for a causal effect of pair‐bond duration on reproductive performance in oystercatchers (*Haematopus ostralegus*). Behavioral Ecology, 17, 982–991. 10.1093/beheco/arl036

[ece370081-bib-0117] Wiley, E. M. , & Ridley, A. R. (2018). The benefits of pair bond tenure in the cooperatively breeding pied babbler (*Turdoides bicolor*). Ecology and Evolution, 8, 7178–7185. 10.1002/ece3.4243 30073076 PMC6065330

[ece370081-bib-0118] Williams, H. J. , Shepard, E. L. C. , Holton, M. D. , Alarcón, P. A. E. , Wilson, R. P. , & Lambertucci, S. A. (2020). Physical limits of flight performance in the heaviest soaring bird. Proceedings of the National Academy of Sciences of the United States of America, 117, 17884–17890. 10.1073/pnas.1907360117 32661147 PMC7395523

[ece370081-bib-0119] Wilmers, C. C. , Nickel, B. , Bryce, C. M. , Smith, J. A. , Wheat, R. E. , & Yovovich, V. (2015). The golden age of bio‐logging: How animal‐borne sensors are advancing the frontiers of ecology. Ecology, 96, 1741–1753. 10.1890/14-1401.1 26378296

