# Supplementary material for: Both movements and breeding performance are affected by individual experience in the Bonelli's eagle Aquila fasciata
Source: Ecol Evol. 2024 Jul 24;14(7):e70081. doi: 10.1002/ece3.70081 (PMC11268896; doi:10.1002/ece3.70081)
Supplement: Supplementary file 1 — Appendix S1 [file ECE3-14-e70081-s001.pdf]

Ecology & Evolution - Supplementary materials

# Both movements and breeding performance are affected by individual experience in the Bonelli's eagle *Aquila fasciata*

Lise Viollat, Alexandre Millon, Cécile Ponchon, Alain Ravayrol, Thibaut Couturier, Aurélien Besnard

APPENDIX S1: Breeding performances, experience and recruitment in the French population of Bonelli’s eagle

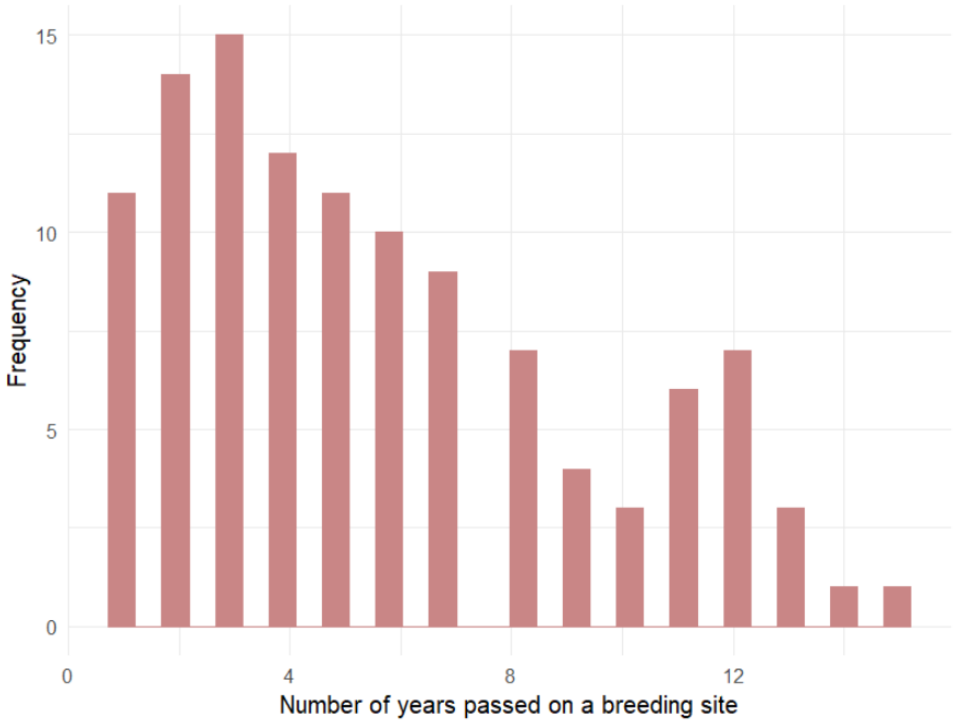

Figure S1-1: Distribution of individual experience, i.e. the number of years spent on a breeding site of Bonelli’s eagles equipped with a GPS tag (48 individuals, 114 breeding events).

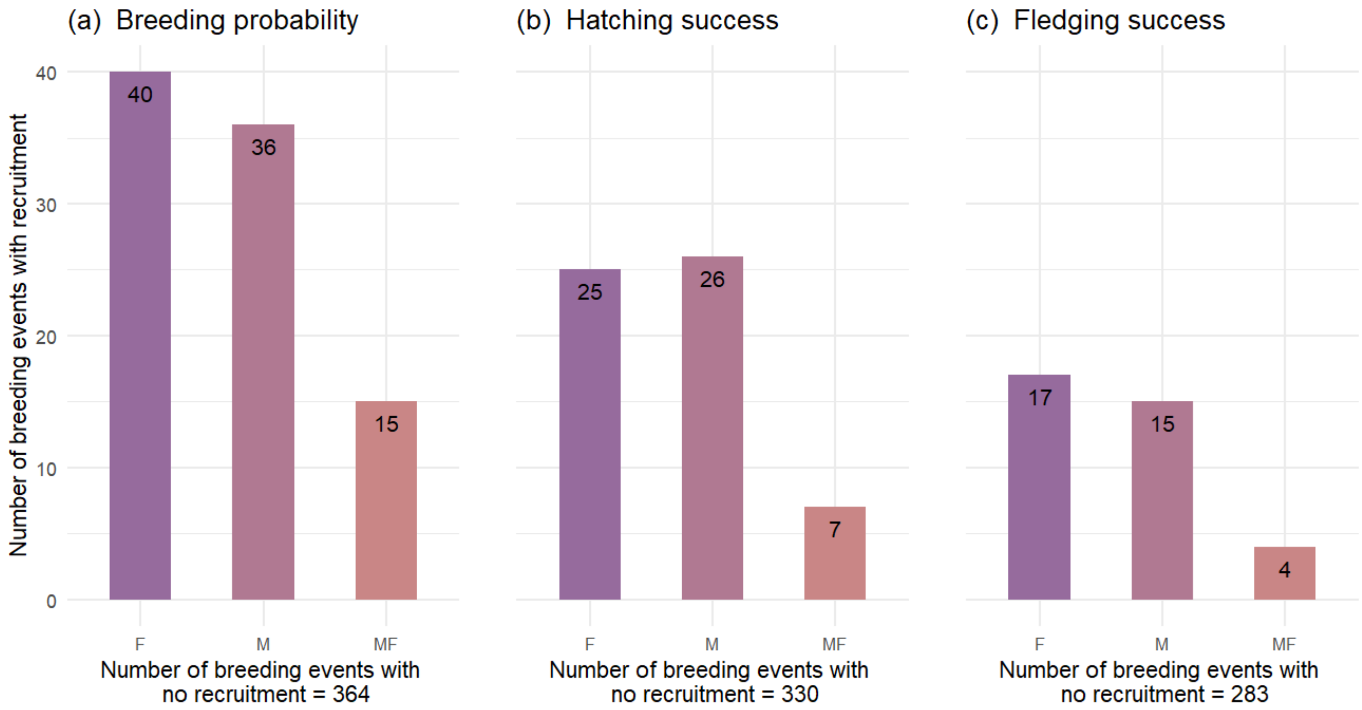

Figure S2-2: The columns show the number of breeding events with the recruitment of a female (F), of a male (M) or of both (MF) for (a) pairs having laid among the pairs present on breeding sites (breeding probability), (b) pairs having at least one hatching among the pairs having laid eggs (hatching success) and (c) pairs having fledged at least one young among pairs having hatched at least one egg (fledging success). As a means of comparison, the number of breeding events without any recruitment is indicated in the black text below each graph. The data come from the monitoring of all known breeding sites of the French population of Bonelli's eagles between 2008 and 2022.

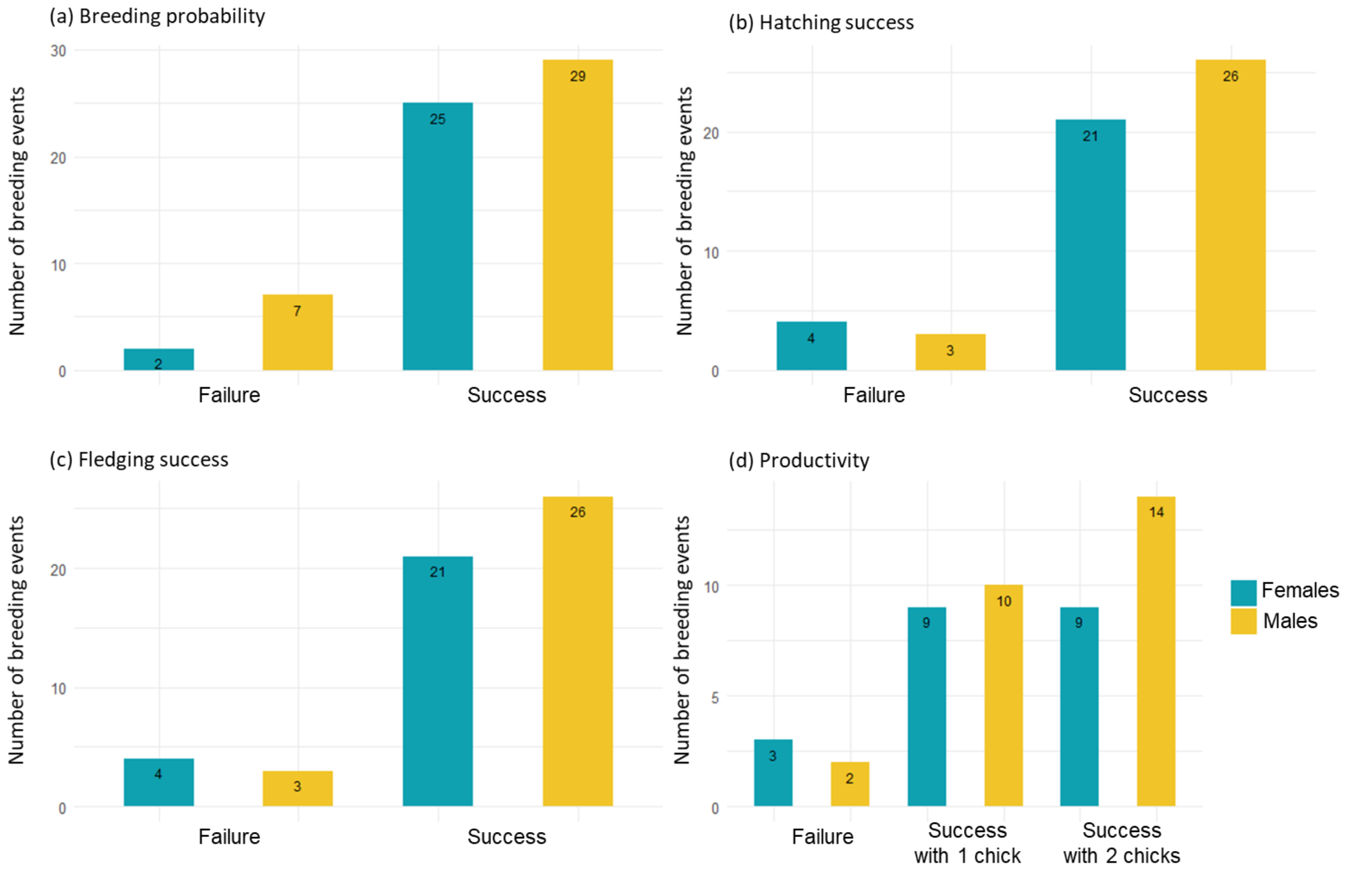

Figure S1-3: Number of successes and failures in (a) laying, (b) hatching, (c) fledging, and the (d) number of fledging chicks observed in the French population of Bonelli's eagle for the individuals equipped with GPS tags (n=11 females and 15 males for 63 breeding events), separated by females (in blue) and males (in yellow).
