# Supplementary material for: Both movements and breeding performance are affected by individual experience in the Bonelli's eagle Aquila fasciata
Source: Ecol Evol. 2024 Jul 24;14(7):e70081. doi: 10.1002/ece3.70081 (PMC11268896; doi:10.1002/ece3.70081)
Supplement: Supplementary file 2 — Appendix S2 [file ECE3-14-e70081-s005.pdf]

Ecology & Evolution - Supplementary materials

# Both movements and breeding performance are affected by individual experience in the Bonelli's eagle *Aquila fasciata*

Lise Viollat, Alexandre Millon, Cécile Ponchon, Alain Ravayrol, Thibaut Couturier, Aurélien Besnard

APPENDIX S2: Distribution of weather covariates during the breeding season

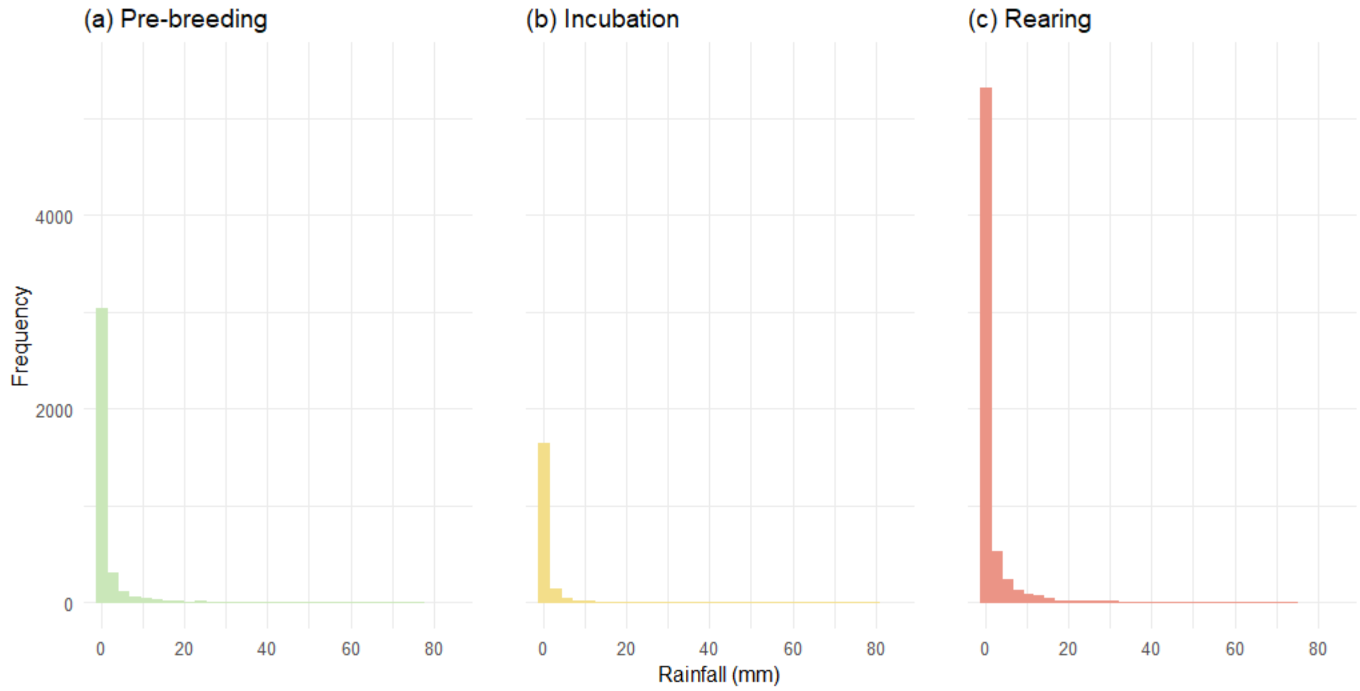

Figure S2-1: Daily rainfall (mm) during (a) pre-breeding, (b) incubation and (c) rearing inside the home range of Bonelli’s eagles equipped with GPS tags.

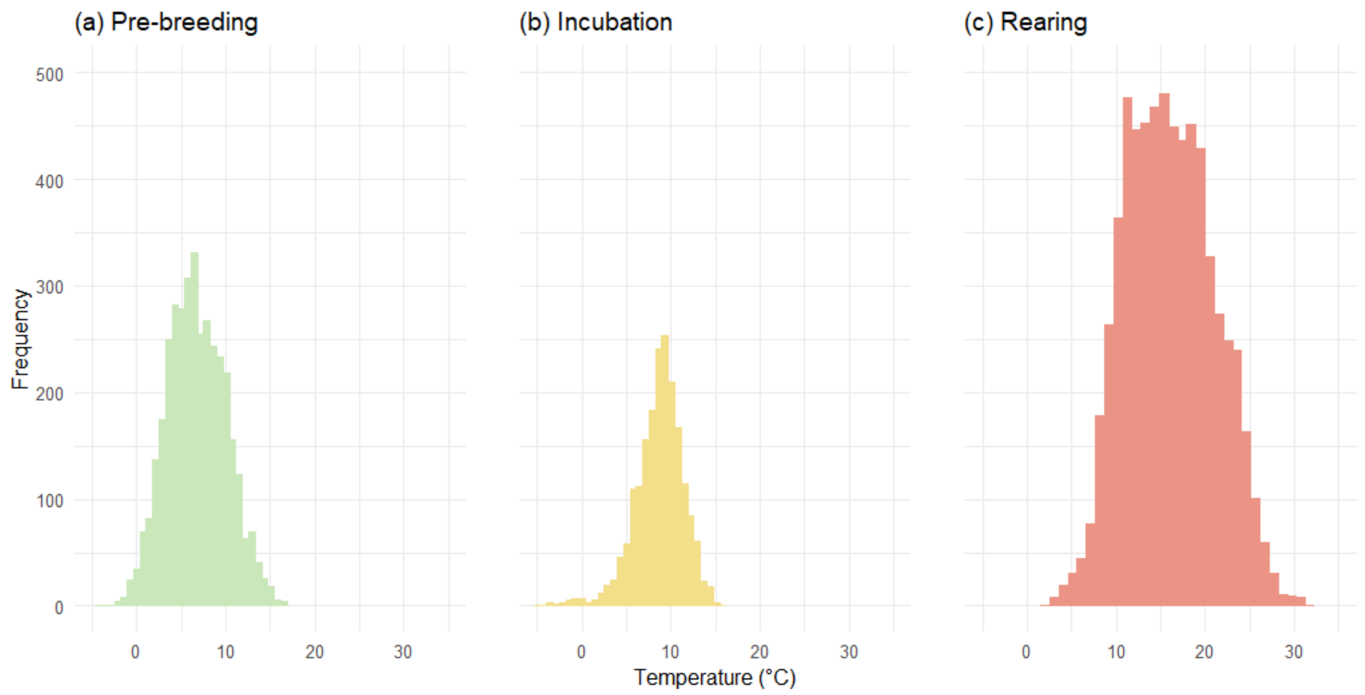

Figure S2-2: Daily mean temperature (°C) during (a) pre-breeding, (b) incubation and (c) rearing inside the home range of Bonelli's eagles equipped with GPS tags.

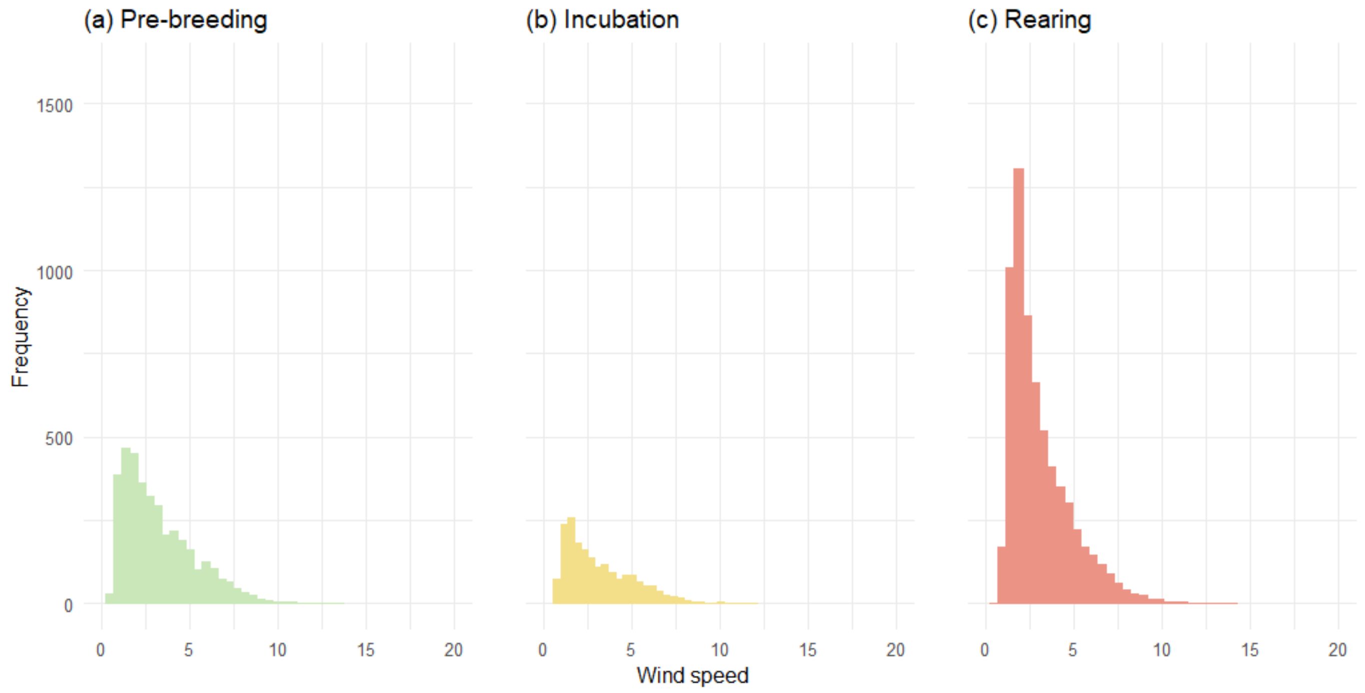

Figure S2-3: Daily wind speed (in $m.s^{-1}$) during (a) pre-breeding, (b) incubation and (c) rearing inside the home range of Bonelli's eagles equipped with GPS tags.

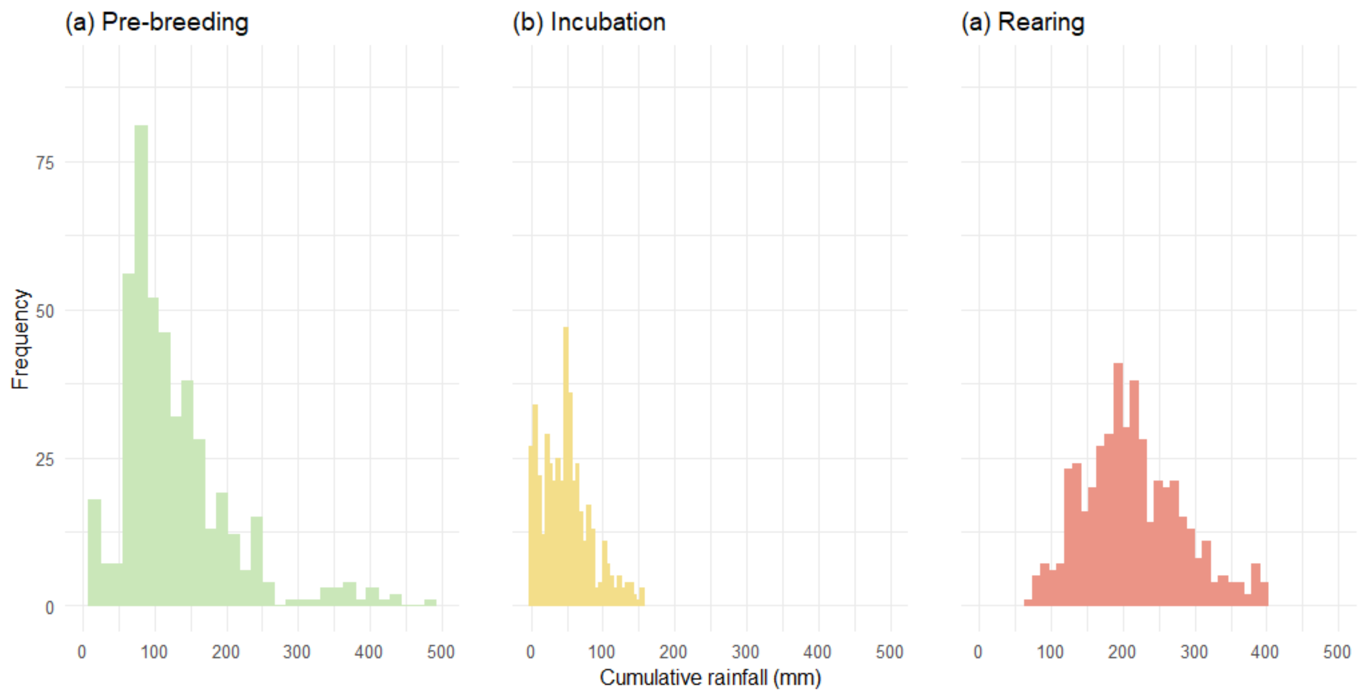

Figure S2-4: Cumulative rainfall (mm) during (a) pre-breeding, (b) incubation and (c) rearing, for all known breeding sites of the French population of Bonelli's eagle, between 2009 and 2022.

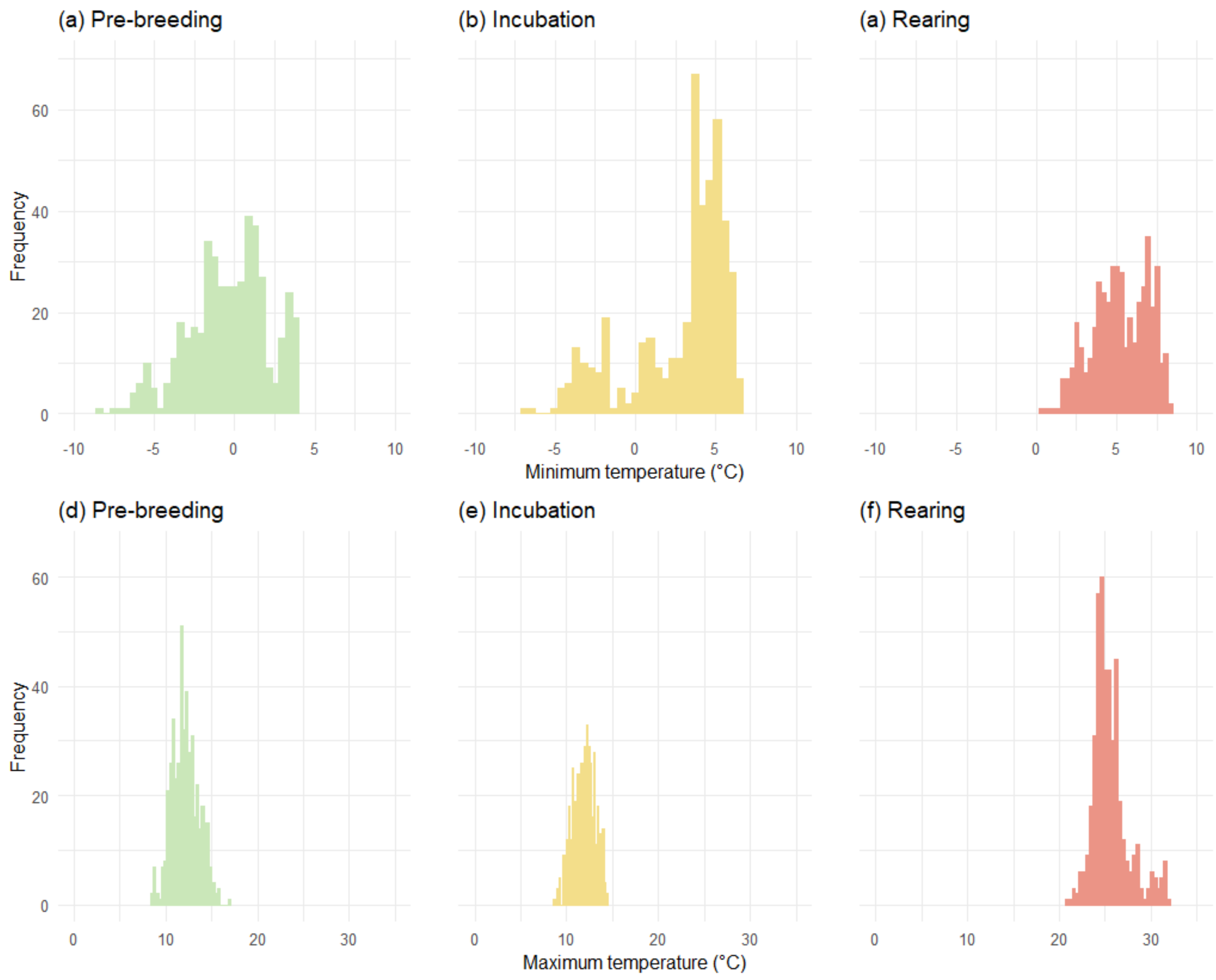

Figure S2-5: Minimal temperature (°C) during (a) pre-breeding, (b) incubation and (c) rearing; and maximum temperature (°C) during (d) pre-breeding, (e) incubation and (f) rearing, for all known breeding sites of the French population of Bonelli's eagle, between 2009 and 2022.

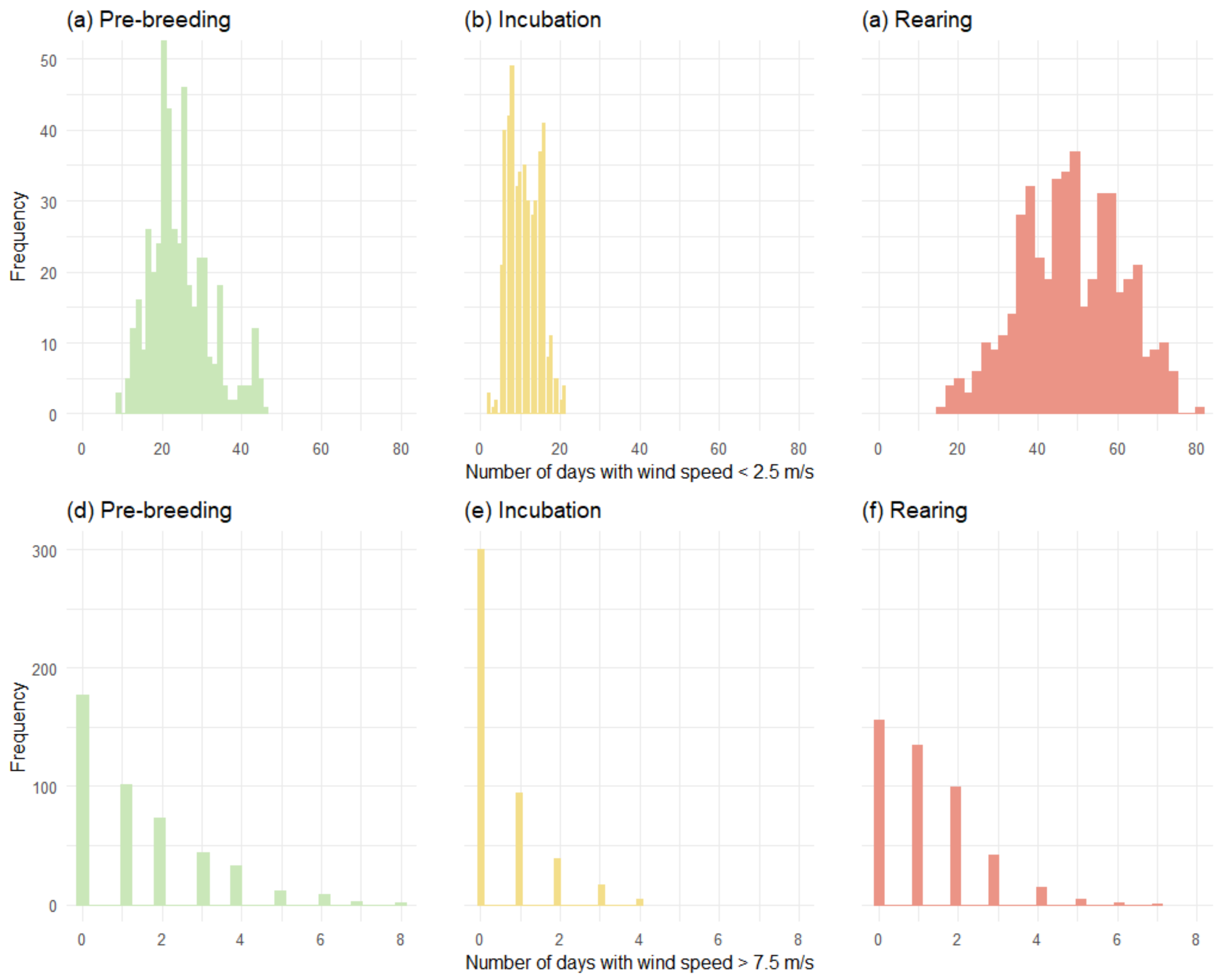

Figure S2-6: Number of days with wind speed under 2.5 $m.s^{-1}$ during (a) pre-breeding, (b) incubation and (c) rearing; and number of days with wind speed above 7.5 $m.s^{-1}$ during (d) pre-breeding, (e) incubation and (f) rearing, for all known breeding sites of the French population of Bonelli's eagle, between 2009 and 2022.
