# Supplementary material for: Both movements and breeding performance are affected by individual experience in the Bonelli's eagle Aquila fasciata
Source: Ecol Evol. 2024 Jul 24;14(7):e70081. doi: 10.1002/ece3.70081 (PMC11268896; doi:10.1002/ece3.70081)
Supplement: Supplementary file 3 — Appendix S3 [file ECE3-14-e70081-s003.pdf]

Ecology & Evolution - Supplementary materials

# Both movements and breeding performance are affected by individual experience in the Bonelli's eagle *Aquila fasciata*

Lise Viollat, Alexandre Millon, Cécile Ponchon, Alain Ravayrol, Thibaut Couturier, Aurélien Besnard

APPENDIX S3: Individual variability in movement behaviour

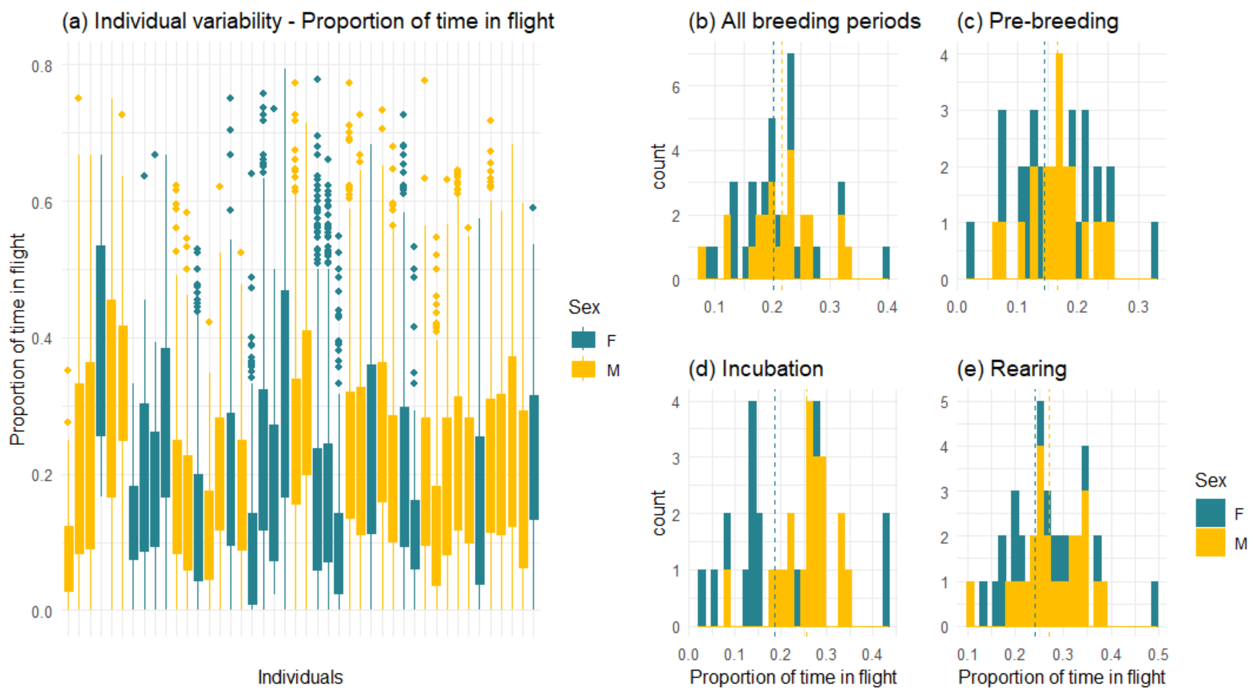

Figure S3-1: (a) Individual variability of the daily proportion of time in flight during the breeding season, and mean individual proportion of time in flight for all breeding periods (b), pre-breeding (c), incubation (d) and rearing (e) for Bonelli’s eagles equipped with GPS tags (n=48). Females are indicated in blue and males in yellow. The dashed lines represent the mean for all the females and all the males of the French population.

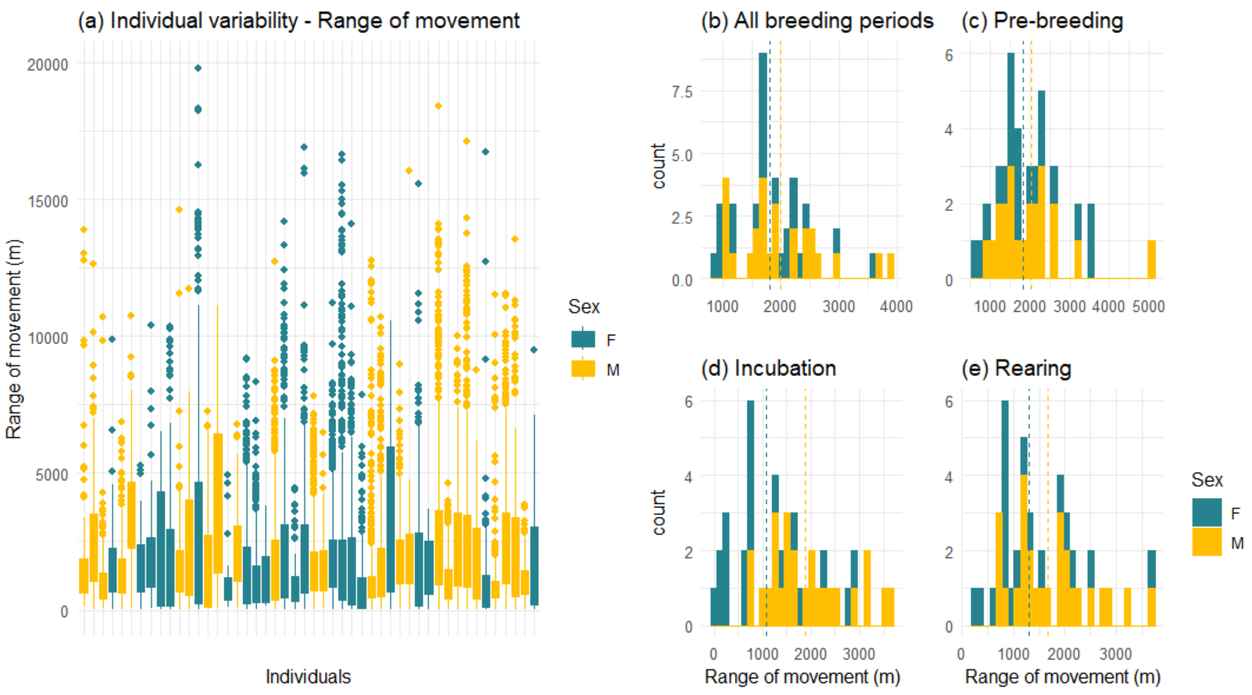

Figure S3-2: (a) Individual variability of the daily range of movement during the breeding season and mean individual range of movement for all breeding periods (b), pre-breeding (c), incubation (d) and rearing (e) for Bonelli's eagles equipped with GPS tags (n=48). Females are indicated in blue and males in yellow. The dashed lines represent the mean for all the females and all the males of the French population.

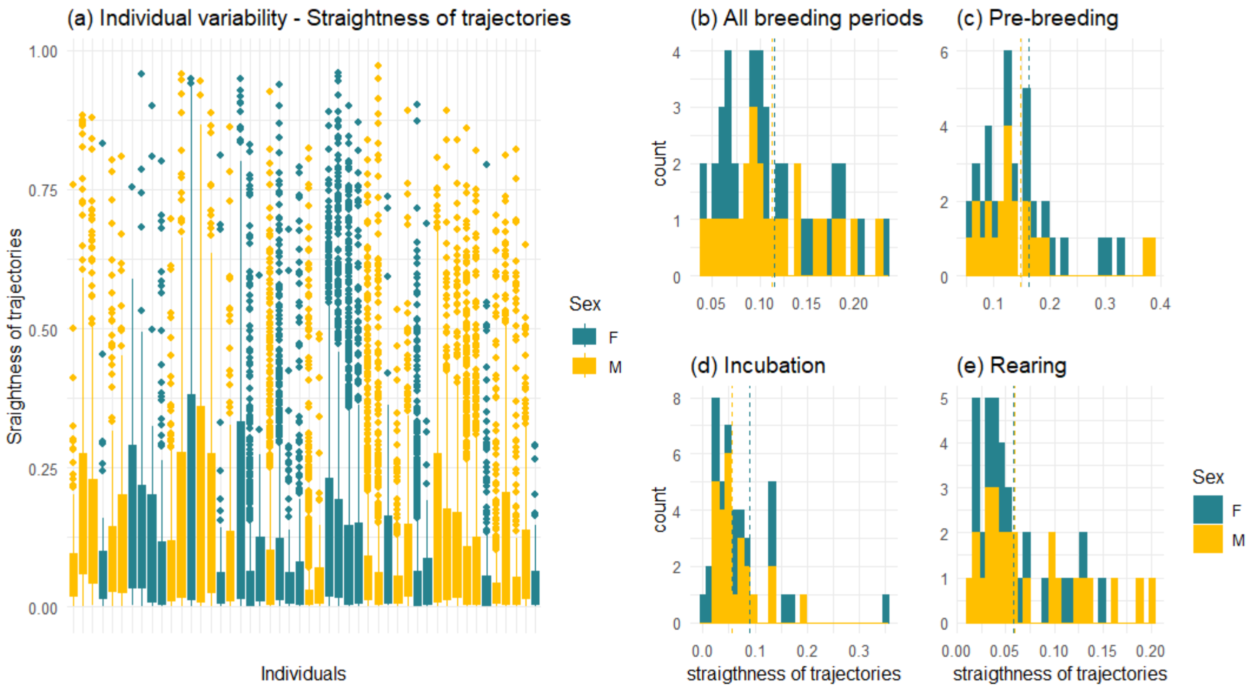

Figure S3-3: (a) Individual variability of the daily straightness of trajectories during the breeding season and mean individual straightness of trajectories in flight for all breeding periods (b), pre-breeding (c), incubation (d) and rearing (e) for Bonelli's eagles equipped with GPS tags (n=48). Females are indicated in blue and males in yellow. The dashed lines represent the mean for all the females and all the males of the French population.
