# Supplementary material for: Both movements and breeding performance are affected by individual experience in the Bonelli's eagle Aquila fasciata
Source: Ecol Evol. 2024 Jul 24;14(7):e70081. doi: 10.1002/ece3.70081 (PMC11268896; doi:10.1002/ece3.70081)
Supplement: Supplementary file 4 — Appendix S4 [file ECE3-14-e70081-s004.pdf]

Ecology & Evolution - Supplementary materials

# Both movements and breeding performance are affected by individual experience in the Bonelli's eagle *Aquila fasciata*

Lise Viollat, Alexandre Millon, Cécile Ponchon, Alain Ravayrol, Thibaut Couturier, Aurélien Besnard

APPENDIX S4: Effect of wind on movements of Bonelli's eagles

Only significant relationships are represented here.

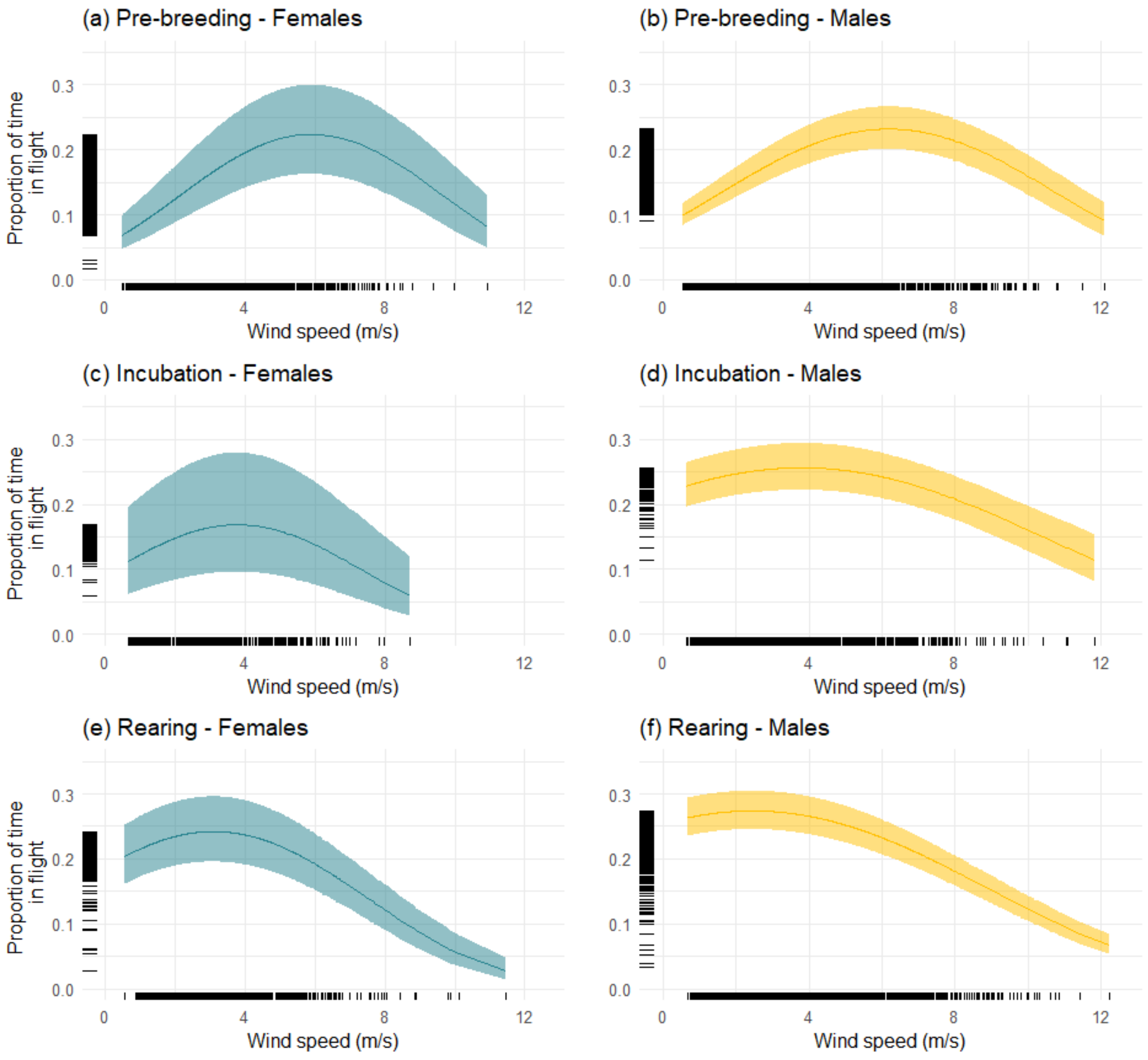

Figure S4-1: Effect of wind speed on the proportion of time in flight during pre-breeding for females (a) and males (c), during incubation (c) for females and (d) males, and during rearing for (e) females and (f) males.

Solid line represents the modelled relationship obtained from the covariate model, and shaded areas represent the 95% credibility intervals. Males are represented in yellow and females in blue.

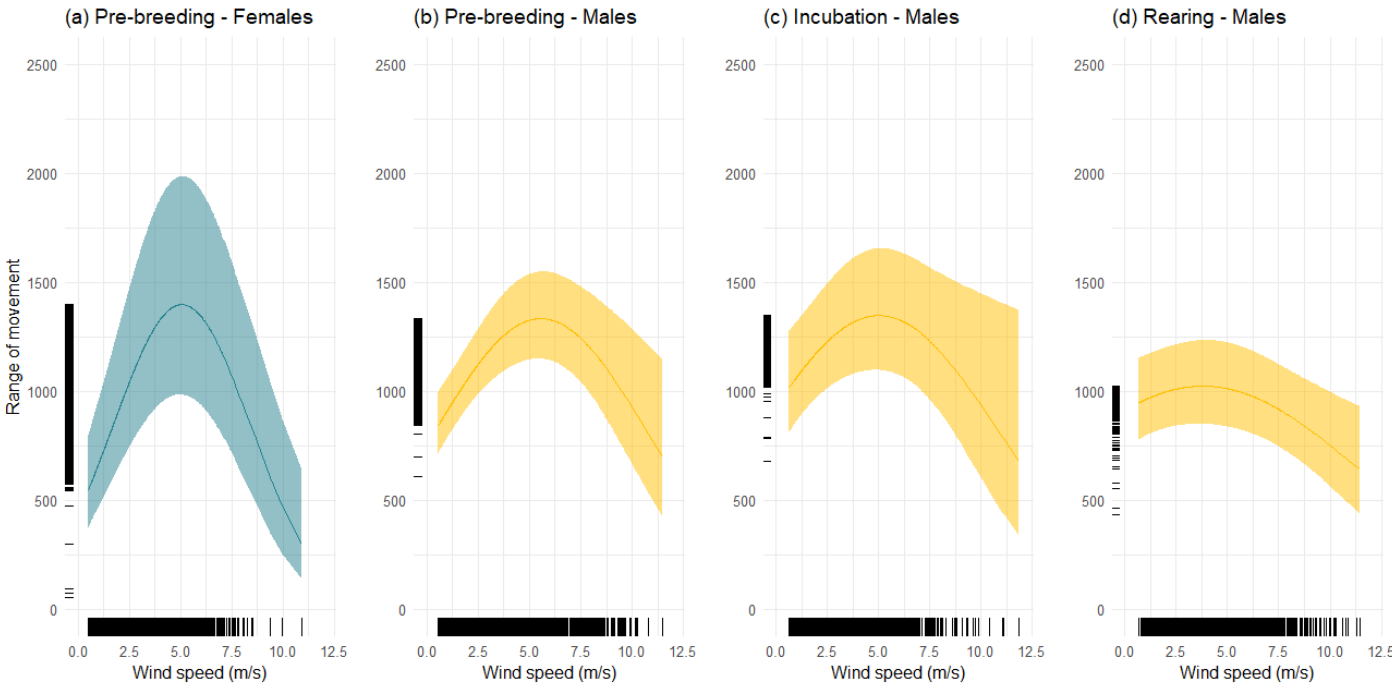

Figure S4-2: Effect of wind speed on the range of movement during pre-breeding for females (a) and males (c), and during (c) incubation and (d) rearing for males. Solid line represents the modelled relationship obtained from the covariate model, and shaded areas represent the 95% credibility intervals. Males are represented in yellow and females in blue.

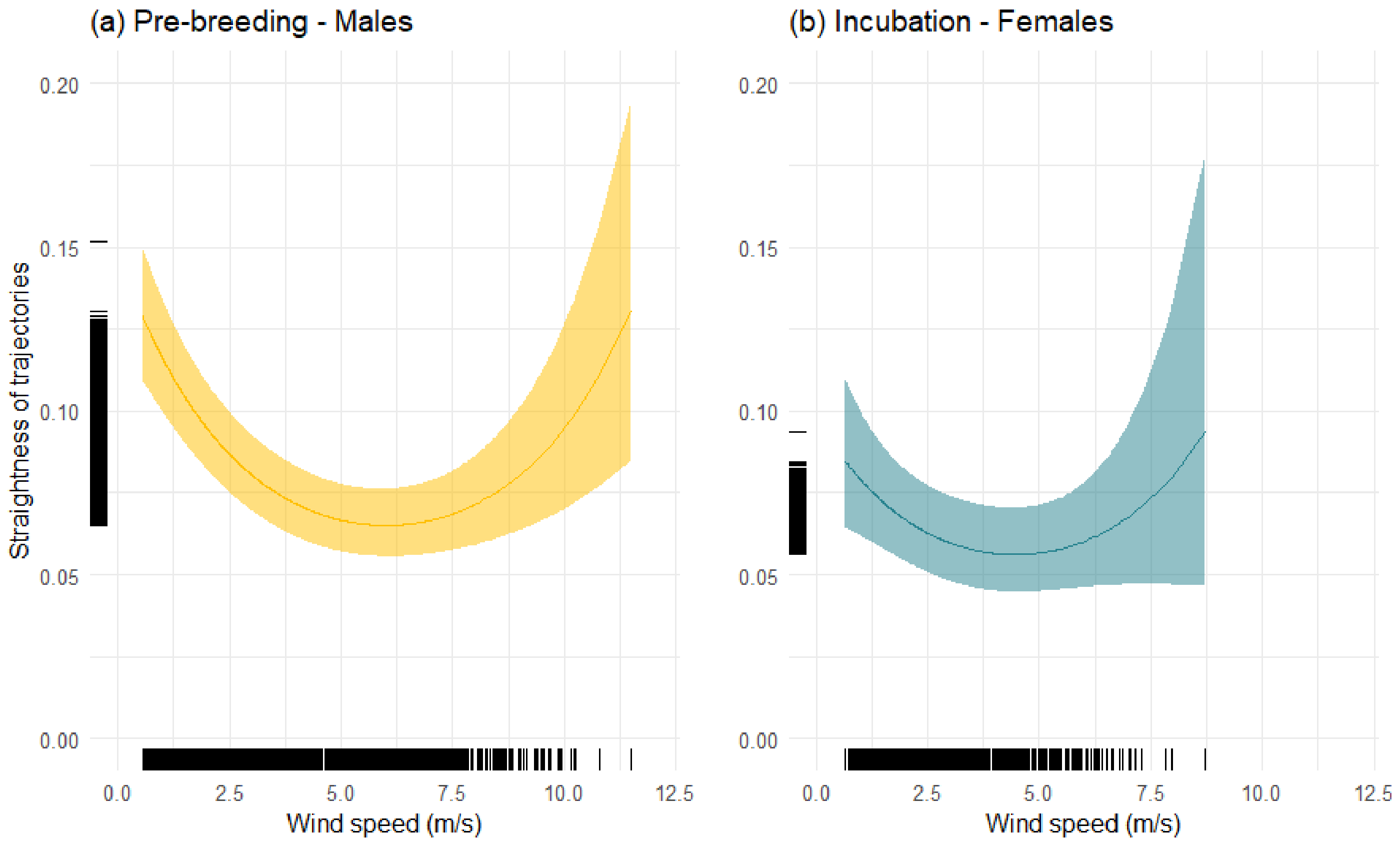

Figure S4-3: Effect of wind speed on the straightness of trajectories during pre-breeding for males (a) and during incubation for females (b). Solid line represents the modelled relationship obtained from the covariate model, and shaded areas represent the 95% credibility intervals. Males are represented in yellow and females in blue.
