# Supplementary material for: Both movements and breeding performance are affected by individual experience in the Bonelli's eagle Aquila fasciata
Source: Ecol Evol. 2024 Jul 24;14(7):e70081. doi: 10.1002/ece3.70081 (PMC11268896; doi:10.1002/ece3.70081)
Supplement: Supplementary file 5 — Appendix S5 [file ECE3-14-e70081-s002.pdf]

# Both movements and breeding performance are affected by individual experience in the Bonelli's eagle *Aquila fasciata*

Lise Viollat, Alexandre Millon, Cécile Ponchon, Alain Ravayrol, Thibaut Couturier, Aurélien Besnard

APPENDIX S5: Model coefficients

For each model, all continuous explanatory variables were standardized as $Z_i = (X_i - \bar{X})/\sigma$ (with Z the standardized variable, X the original variable and σ its standard deviation) to allow the comparison of the different effect sizes among each other. Correlations between variables (Pearson correlation coefficient > 0.6) were verified.

	Response variable	Phase	Intrinsic & extrinsic factors	Random effect	Observations scales	Number of observations	
**Effect of intrinsic and extrinsic factors on breeding performance**	Breeding probability	Pre-breeding	Recruitment (M, F and MF), rainfall, wind < 2.5 m.s^{-1}, wind > 7.5 m.s^{-1}, Min T°C, Max T°C	Breeding site (n=36)	Breeding event (1 per year) for all territorial individuals		455
	Hatching success	Incubation					381
	Fledging success	Rearing	Recruitment (M and F), rainfall, wind < 2.5 m.s^{-1}, wind > 7.5 m.s^{-1}, Min T°C, Max T°C				315
**Effect of intrinsic and extrinsic factors on movements behaviours**	Proportion of time in flight	Pre-breeding	Experience, Rainfall, T°C, Wind, Wind²	Individual identifier (n= 15 females, 21 males)	Daily behaviours for individuals equipped with GPS tags	Females	1244
						Males	1764
		Incubation				Females	481
						Males	969
		Rearing				Females	1562
						Males	2975
	Range of movement	Pre-breeding	Number of GPS locations, Experience, Rainfall, T°C, Wind, Wind²			Females	1329
						Males	1782
		Incubation				Females	577
						Males	1074
		Rearing				Females	1854
						Males	3358
	Straightness of trajectories	Pre-breeding				Females	1428
						Males	1887
		Incubation				Females	631
						Males	1089
		Rearing				Females	2117
						Males	3575
**Relationship between movement behaviours and productivity**	Productivity	Pre-breeding	Averaged (for the phase) proportion of time in flight, range of movement and straightness of trajectories	Individual identifier (n= 11 females, 15 males)	Breeding event (1 per year) for individuals equipped with GPS tags	Females	27
						Males	36
		Incubation				Females	25
						Males	29
		Rearing				Females	21
						Males	26

Table S5-1: Summary of the different models fitted to investigate the relationships between different measures of breeding performance (breeding probability, hatching success, fledging success) and

productivity, movement behaviours (proportion of time in flight, range of movement, straightness of trajectories), intrinsic (recruitment and experience) and extrinsic factors (local weather: rainfall, number of days with wind speed above 7.5 m.s-1 [wind > 7.5 m.s-1] or below 2.5 m.s-1 [wind < 2.5 m.s-1], minimum [min T°C], maximum [max T°C], or mean temperature [T°C], and wind speed [Wind and Wind²]).

Section S5-1: EFFECT OF INTRINSIC AND EXTRINSIC FACTORS ON BREEDING SUCCESS

Table S5-2: Table of estimated slope, 95% confidence intervals (CI) and p-value for the effect of recruitment (F: recruitment of a female, M: recruitment of a male, MF: recruitment of both individuals on a breeding site), cumulative rainfall (in mm), minimum and maximum mean daily temperature (min and max T°C), and the number of days with wind speed above 7.5 $m.s^{-1}$ and below 2.5 $m.s^{-1}$ during the pre-breeding period on breeding probability, i.e. pairs having laid among the pairs present on breeding sites (binomial mixed model).

Breeding probability (n=455)

predictor	*Estimated slope*	*Lower CI*	*Upper CI*	*p-value*
Recruitment F	-1.98	-2.88	-1.08	<0.01 ***
Recruitment M	-1.13	-2.09	-0.17	0.02*
Recruitment MF	-3.03	-4.47	-1.59	<0.01***
Cumulative rainfall	0.00	-0.39	0.39	0.70
Wind speed < 2.5 $m.s^{-1}$	-0.08	-0.51	0.34	0.71
Wind speed > 7.5 $m.s^{-1}$	-0.07	-0.45	0.31	0.99
Min T°C	0.35	-0.08	0.79	0.11
Max T°C	-0.18	-0.60	0.23	0.39

*: p-value between 0.05 and 0.01, **: p-value between 0.01 and 0.001, ***: p-value < 0.001, . : p-value between 0.05 and 0.1

Table S5-3: Table of estimated slope, 95% confidence intervals (CI) and p-value for the effect of recruitment (F: recruitment of a female, M: recruitment of a male), cumulative rainfall (in mm), minimum and maximum mean daily temperature (min and max T°C), and the number of days with wind speed above 7.5 $m.s^{-1}$ and below 2.5 $m.s^{-1}$ during the incubation period on hatching success, i.e. pairs having at least one hatching among the pairs having laid eggs (binomial mixed model).

Hatching success (n=381)

predictor	*Estimated slope*	*Lower CI*	*Upper CI*	*p-value*
Recruitment F	-0.98	-1.98	0.01	0.05 .
Recruitment M	-1.62	-2.56	-0.68	<0.01***
Cumulative rainfall	0.01	-0.39	0.40	0.97

Wind speed < 2.5 m.s^{-1}	0.10	-0.27	0.48	0.59
Wind speed > 7.5 m.s^{-1}	-0.07	-0.39	0.23	0.62
Min T°C	-0.02	-0.39	0.34	0.88
Max T°C	-0.30	-0.64	0.04	0.08 .

*: p-value between 0.05 and 0.01, **: p-value between 0.01 and 0.001, ***: p-value < 0.001, . : p-value between 0.05 and 0.1

Table S5-4: Table of estimated slope, 95% confidence intervals (CI) and p-value for the effect of recruitment (F: recruitment of a female, M: recruitment of a male), cumulative rainfall (in mm), minimum and maximum mean daily temperature (min and max T°C), and the number of days with wind speed above 7.5 m.s^{-1} and below 2.5 m.s^{-1} during the rearing period on fledging success, i.e. pairs having fledged at least one young among pairs having hatched at least one egg (binomial mixed model).

Fledging success (n=315)

predictor	*Estimated slope*	*Lower CI*	*Upper CI*	*p-value*
Recruitment F	-1.29	-2.59	0.01	0.05 .
Recruitment M	0.66	-1.96	2.33	0.17
Cumulative rainfall	-0.20	-0.70	0.29	0.42
Wind speed < 2.5 m.s^{-1}	-0.13	-0.59	0.31	0.55
Wind speed > 7.5 m.s^{-1}	0.30	-0.22	0.83	0.25
Min T°C	[illegible]	[illegible]	[illegible]	[illegible]
Max T°C	-0.48	-0.94	-0.01	0.04*

**: p-value between 0.05 and 0.01, **: p-value between 0.01 and 0.001, ***: p-value between 0.001 and 0, -: p-value > 0.05*

*: p-value between 0.05 and 0.01, **: p-value between 0.01 and 0.001, ***: p-value < 0.001, . : p-value between 0.05 and 0.1

Section S5-2: EFFECT OF INTRINSIC AND EXTRINSIC FACTORS ON MOVEMENT BEHAVIOUR

Proportion of time in flight

Table S5-5: Table of estimated slope (E slope), 95% confidence intervals (CI) and p-value for the effect of an individual's experience (number of years spent on the breeding site since recruitment) and local weather (rainfall, temperature [T°C], and wind speed [Wind and Wind²]) on the proportion of time in flight during pre-breeding, for males and females, at a daily scale (binomial mixed models).

Pre-breeding (n=1244 for females, n=1764 for males)

	Males				Females			
Predictor	*E slope*	*Lower CI*	*Upper CI*	*p-value*	*E slope*	*Lower CI*	*Upper CI*	*p-value*
Experience	0.04	-0.03	0.11	0.29	-0.31	-0.39	-0.22	<0.01***
Rainfall	-0.19	-0.22	-0.15	<0.01***	-0.26	-0.30	-0.21	<0.01***

T°C	0.02	-0.04	0.00	0.05 .	-0.00	-0.03	0.02	0.88
Wind speed	0.80	0.72	0.89	<0.01***	1.11	0.99	1.23	<0.01***
Wind speed ²	-0.58	-0.66	-0.50	<0.01***	-0.87	-1.01	-0.73	<0.01***

*: p-value between 0.05 and 0.01, **: p-value between 0.01 and 0.001, ***: p-value < 0.001, . : p-value between 0.05 and 0.1

Table S5-6: Table of estimated slope (E slope), 95% confidence intervals (CI) and p-value for the effect of an individual's experience (number of years spent on the breeding site since recruitment) and local weather (rainfall, temperature [T°C], and wind speed [Wind and Wind²]) on the proportion of time in flight during incubation, for males and females, at a daily scale (binomial mixed models).

Incubation (n=481 for females, n=969 for males)

	Males				Females			
Predictor	*E slope*	*Lower CI*	*Upper CI*	*p-value*	*E slope*	*Lower CI*	*Upper CI*	*p-value*
Experience	-0.14	-0.22	-0.05	<0.01***	-0.70	-0.84	-0.55	<0.01***
Rainfall	-0.21	-0.25	-0.17	<0.01***	-0.28	-0.37	-0.18	<0.01***
T°C	0.09	0.07	0.12	<0.01***	0.05	0.01	0.09	0.01*
Wind speed	0.23	0.14	0.32	<0.01***	0.65	0.45	0.85	<0.01***
Wind speed ²	-0.27	-0.36	-0.18	<0.01***	-0.70	-0.93	-0.47	<0.01***

*: p-value between 0.05 and 0.01, **: p-value between 0.01 and 0.001, ***: p-value < 0.001, . : p-value between 0.05 and 0.1

Table S5-7: Table of estimated slope (E slope), 95% confidence intervals (CI) and p-value for the effect of an individual's experience (number of years spent on the breeding site since recruitment) and local weather (rainfall, temperature [T°C], and wind speed [Wind and Wind²]) on the proportion of time in flight during rearing, for males and females, at a daily scale (binomial mixed models).

Rearing (n=1562 for females, n=2975 for males)

	Males				Females			
Predictor	*E slope*	*Lower CI*	*Upper CI*	*p-value*	*E slope*	*Lower CI*	*Upper CI*	*p-value*
Experience	-0.08	-0.12	-0.03	<0.01***	-0.19	-0.25	-0.13	<0.01***
Rainfall	-0.25	-0.27	-0.23	<0.01***	-0.16	-0.18	-0.13	<0.01***
T°C	-0.06	-0.07	-0.04	<0.01***	0.17	0.15	0.19	<0.01***
Wind speed	0.15	0.11	0.20	<0.01***	0.34	0.25	0.44	<0.01***
Wind speed ²	-0.30	-0.35	-0.25	<0.01***	-0.48	-0.59	-0.36	<0.01***

*: p-value between 0.05 and 0.01, **: p-value between 0.01 and 0.001, ***: p-value < 0.001, . : p-value between 0.05 and 0.1

Range of movement

Table S5-8: Table of estimated slope (E slope), 95% confidence intervals (CI) and p-value for the effect of the number of GPS locations (n GPS loc), an individual's experience (number of years spent on the breeding site since recruitment) and local weather (rainfall, temperature [T°C], and wind speed [Wind and Wind²]) on the range of movement during pre-breeding, for males and females, at a daily scale (Gaussian mixed models).

Pre-breeding (n=1329 for females, n=1782 for males)

	Males				Females			
Predictor	*E slope*	*Lower CI*	*Upper CI*	*p-value*	*E slope*	*Lower CI*	*Upper CI*	*p-value*
n GPS loc	-0.02	-0.08	0.03	0.36	0.31	0.16	0.47	<0.01***
Experience	0.04	-0.05	0.15	0.43	-0.45	-0.65	-0.25	<0.01***
Rainfall	-0.11	-0.16	-0.06	<0.01***	-0.16	-0.23	-0.09	<0.01***
T°C	0.02	-0.03	0.07	0.40	-0.05	-0.12	0.02	0.18
Wind speed	0.42	0.24	0.59	<0.01***	0.90	0.66	1.14	<0.01***
Wind speed ²	-0.33	-0.51	-0.16	<0.01***	-0.81	-1.06	-0.56	<0.01***

*: p-value between 0.05 and 0.01, **: p-value between 0.01 and 0.001, ***: p-value < 0.001, . : p-value between 0.05 and 0.1

Table S5-9: Table of estimated slope (E slope), 95% confidence intervals (CI) and p-value for the effect of the number of GPS locations (n GPS loc), an individual's experience (number of years spent on the breeding site since recruitment) and local weather (rainfall, temperature [T°C], and wind speed [Wind and Wind²]) on the range of movement during incubation, for males and females, at a daily scale (Gaussian mixed models).

Incubation (n= 577 for females, n= 1074 for males)

	Males				Females			
Predictor	*E slope*	*Lower CI*	*Upper CI*	*p-value*	*E slope*	*Lower CI*	*Upper CI*	*p-value*
n GPS loc	0.10	0.01	0.19	0.02*	0.29	0.13	0.45	<0.01***
Experience	-0.12	-0.27	0.02	0.09 .	-0.77	-1.05	-0.49	<0.01***
Rainfall	-0.03	-0.09	0.01	0.17	-0.13	-0.23	-0.03	<0.01**
T°C	0.007	-0.05	0.07	0.81	-0.05	-0.15	0.04	0.25
Wind speed	0.29	0.08	0.49	<0.01**	0.20	-0.24	0.65	0.37
Wind speed ²	-0.26	-0.46	-0.05	0.01*	-0.23	-0.73	0.25	0.34

*: p-value between 0.05 and 0.01, **: p-value between 0.01 and 0.001, ***: p-value < 0.001, . : p-value between 0.05 and 0.1

Table S5-10: Table of estimated slope (E slope), 95% confidence intervals (CI) and p-value for the effect of the number of GPS locations (n GPS loc), an individual's experience (number of years spent on the breeding

site since recruitment) and local weather (rainfall, temperature [T°C], and wind speed [Wind and Wind²]) on the range of movement during rearing, for males and females, at a daily scale (Gaussian mixed models).

Rearing (n=1854 for females, n=3358 for males)

	Males				Females			
Predictor	*E slope*	*Lower CI*	*Upper CI*	*p-value*	*E slope*	*Lower CI*	*Upper CI*	*p-value*
n GPS loc	0.01	-0.22	-0.01	0.65	0.22	0.11	0.32	<0.01***
Experience	-0.12	-0.05	0.08	0.02*	-0.56	-0.73	-0.39	<0.01***
Rainfall	-0.08	-0.12	-0.05	<0.01***	-0.01	-0.07	0.05	0.73
T°C	0.02	-0.01	0.05	0.23	0.54	0.47	0.61	<0.01***
Wind speed	0.11	0.001	0.22	0.04*	0.20	-0.03	0.43	0.09 .
Wind speed ²	-0.13	-0.24	-0.02	0.01*	-0.10	-0.35	0.14	0.42

*: p-value between 0.05 and 0.01, **: p-value between 0.01 and 0.001, ***: p-value < 0.001, . : p-value between 0.05 and 0.1

Straightness of trajectories

Table S5-11: Table of estimated slope (E slope), 95% confidence intervals (CI) and p-value for the effect of the number of GPS locations (n GPS loc), an individual's experience (number of years spent on the breeding site since recruitment) and local weather (rainfall, temperature [T°C], and wind speed [Wind and Wind²]) on the straightness of trajectories during pre-breeding, for males and females, at a daily scale (Gaussian mixed models).

Pre-breeding (n=1428 for females, n=1887 for males)

	Males				Females			
Predictor	*E slope*	*Lower CI*	*Upper CI*	*p-value*	*E slope*	*Lower CI*	*Upper CI*	*p-value*
n GPS loc	-0.37	-0.43	-0.31	<0.01***	-0.26	-0.40	-0.11	<0.01***
Experience	0.02	-0.09	0.14	0.65	-0.19	-0.36	-0.01	0.03*
Rainfall	0.08	0.03	0.12	<0.01***	0.10	0.03	0.17	<0.01**
T°C	0.05	0.00	0.10	0.04*	0.07	0.00	0.14	0.04*
Wind speed	-0.62	-0.79	-0.45	<0.01***	-0.41	-0.64	-0.17	<0.01***
Wind speed ²	0.46	0.30	0.63	<0.01***	0.16	-0.07	0.41	0.18

*: p-value between 0.05 and 0.01, **: p-value between 0.01 and 0.001, ***: p-value < 0.001, . : p-value between 0.05 and 0.1

Table S5-12: Table of estimated slope (E slope), 95% confidence intervals (CI) and p-value for the effect of the number of GPS locations (n GPS loc), an individual's experience (number of years spent on the breeding site since recruitment) and local weather (rainfall, temperature [T°C], and wind speed [Wind and Wind²]) on

the straightness of trajectories during incubation, for males and females, at a daily scale (Gaussian mixed models).

Incubation (n=631 for females, n=1089 for males)

	Males				Females			
Predictor	*E slope*	*Lower CI*	*Upper CI*	*p-value*	*E slope*	*Lower CI*	*Upper CI*	*p-value*
n GPS loc	-0.19	-0.27	-0.12	<0.01***	-0.15	-0.26	-0.04	<0.01**
Experience	-0.04	-0.16	0.07	0.44	-0.14	-0.31	0.02	0.09 .
Rainfall	0.08	0.03	0.12	<0.01***	0.05	-0.02	0.13	0.14
T°C	-0.00	-0.05	0.04	0.85	-0.02	-0.10	0.05	0.53
Wind speed	-0.08	-0.25	0.08	0.31	-0.47	-0.84	-0.11	0.01*
Wind speed ^{2}	0.13	-0.03	0.30	0.11	0.43	0.03	0.83	0.03*

*: p-value between 0.05 and 0.01, **: p-value between 0.01 and 0.001, ***: p-value < 0.001, .: p-value between 0.05 and 0.1

Table S5-13: Table of estimated slope (E slope), 95% confidence intervals (CI) and p-value for the effect of the number of GPS locations (n GPS loc), an individual's experience (number of years spent on the breeding site since recruitment) and local weather (rainfall, temperature [T°C], and wind speed [Wind and Wind^2]) on the straightness of trajectories during rearing, for males and females, at a daily scale (Gaussian mixed models).

Rearing (n=2117 for females, n=3575 for males)

	Males				Females			
Predictor	*E slope*	*Lower CI*	*Upper CI*	*p-value*	*E slope*	*Lower CI*	*Upper CI*	*p-value*
n GPS loc	-0.31	-0.36	-0.25	<0.01***	-0.13	-0.19	-0.08	<0.01***
Experience	0.00	-0.07	0.08	0.89	-0.12	-0.22	-0.03	<0.01**
Rainfall	0.08	0.06	0.11	<0.01***	0.06	0.02	0.10	<0.01***
T°C	0.02	-0.00	0.04	0.07 .	0.10	0.06	0.14	<0.01***
Wind speed	0.00	-0.07	0.09	0.83	-0.00	-0.14	0.13	0.93
Wind speed ^{2}	0.07	-0.00	0.16	0.07 .	0.13	-0.01	0.29	0.07 .

*: p-value between 0.05 and 0.01, **: p-value between 0.01 and 0.001, ***: p-value < 0.001, . : p-value between 0.05 and 0.1

Section S5-3: RELATIONSHIP BETWEEN MOVEMENT BEHAVIOUR AND PRODUCTIVITY

Table S5-14: Table of estimated slope (E slope), 95% confidence intervals (CI) and p-value for the effect of the proportion of time in flight (% Time in flight), the range of movement and the straightness of trajectories

(straightness) on productivity, i.e. the probability of having 0, 1 or 2 chicks fledging for individuals equipped with GPS tags during pre-breeding, for males and females (Gaussian mixed models).

Pre-breeding (n=27 for females, n=36 for males)

	Males				Females			
Predictor	*E slope*	*Lower CI*	*Upper CI*	*p-value*	*E slope*	*Lower CI*	*Upper CI*	*p-value*
% Time in flight	-0.32	-0.71	0.06	0.10	0.33	-0.12	0.80	0.15
Range of movement	0.11	-0.34	0.57	0.62	-0.18	-0.60	0.24	0.41
Straightness	-0.26	-0.72	0.20	0.26	-0.10	-0.59	0.37	0.66

*: p-value between 0.05 and 0.01, **: p-value between 0.01 and 0.001, ***: p-value < 0.001, . : p-value between 0.05 and 0.1

Table S5-15: Table of estimated slope (E slope), 95% confidence intervals (CI) and p-value for the effect of the proportion of time in flight (% Time in flight), the range of movement and the straightness of trajectories (straightness) on productivity, i.e. the probability of having 0, 1 or 2 chicks fledging for individuals equipped with GPS tags during incubation, for males and females (Gaussian mixed models).

Incubation (n=25 for females, n=29 for males)

	Males				Females			
Predictor	*E slope*	*Lower CI*	*Upper CI*	*p-value*	*E slope*	*Lower CI*	*Upper CI*	*p-value*
% Time in flight	-0.29	-0.62	0.025	0.07 .	-0.23	-0.52	0.05	0.11
Range of movement	0.17	-0.18	0.52	0.34	-0.05	-0.54	0.43	0.81
Straightness	0.00	-0.38	0.38	0.99	-0.41	-0.90	0.08	0.10

*: p-value between 0.05 and 0.01, **: p-value between 0.01 and 0.001, ***: p-value < 0.001, . : p-value between 0.05 and 0.1

Table S5-16: Table of estimated slope (E slope), 95% confidence intervals (CI) and p-value for the effect of the proportion of time in flight (% Time in flight), the range of movement and the straightness of trajectories (straightness) on productivity, i.e. the probability of having 0, 1 or 2 chicks fledging for individuals equipped with GPS tags during rearing, for males and females (Gaussian mixed models).

Rearing (n=21 for females, n=26 for males)

	Males				Females			
Predictor	*E slope*	*Lower CI*	*Upper CI*	*p-value*	*E slope*	*Lower CI*	*Upper CI*	*p-value*
% Time in flight	0.22	-0.51	-0.01	0.03*	0.01	-0.37	0.40	0.93
Range of movement	-0.26	-0.16	0.62	0.26	-0.20	-0.70	0.28	0.40
Straightness	-0.08	-0.41	0.23	0.58	-0.12	-0.54	0.29	0.56

*: p-value between 0.05 and 0.01, **: p-value between 0.01 and 0.001, ***: p-value < 0.001, . : p-value between 0.05 and 0.1

Section S5-4: MOVEMENT BEHAVIOURS BETWEEN SEXES AND PHASES

Table S5-17: Table of estimated slope (E slope), 95% confidence intervals (CI) and p-value for the effect of the sex (Male or females) and of the breeding phases (pre-breeding, incubation, rearing) on the proportion of time in flight for individuals equipped with GPS tags. Sex and phases were considered in interaction on the model (Gaussian mixed models).

Proportion of time in flight (n=111 observations for 44 individuals)

predictor	*Estimated slope*	*Lower CI*	*Upper CI*	*p-value*
Sex M	0.00	-0.04	0.04	0.97
Phase - incubation	0.02	-0.00	0.06	0.15
Phase - rearing	0.09	0.05	0.13	<0.01***
Sex M : incubation	0.06	0.01	0.11	<0.01**
Sex M : rearing	0.01	-0.03	0.05	0.64

*: p-value between 0.05 and 0.01, **: p-value between 0.01 and 0.001, ***: p-value < 0.001, . : p-value between 0.05 and 0.1

Table S5-18: Table of estimated slope (E slope), 95% confidence intervals (CI) and p-value for the effect of the sex (Male or females) and of the breeding phases (pre-breeding, incubation, rearing) on the range of movement for individuals equipped with GPS tags. Sex and phases were considered in interaction on the model (Gaussian mixed models).

Range of movement (n=129 observations for 48 individuals)

predictor	*Estimated slope*	*Lower CI*	*Upper CI*	*p-value*
Sex M	0.11	-0.25	0.49	0.53
Phase - incubation	-1.00	-1.30	-0.69	<0.01***
Phase - rearing	-0.39	-0.71	-0.08	0.01*
Sex M : incubation	0.92	0.51	1.33	<0.01***
Sex M : rearing	0.21	-0.20	0.63	0.31

*: p-value between 0.05 and 0.01, **: p-value between 0.01 and 0.001, ***: p-value < 0.001, . : p-value between 0.05 and 0.1

Table S5-19: Table of estimated slope (E slope), 95% confidence intervals (CI) and p-value for the effect of the sex (Male or females) and of the breeding phases (pre-breeding, incubation, rearing) on the straightness of trajectories for individuals equipped with GPS tags. Sex and phases were considered in interaction on the model (Gaussian mixed models).

Straightness of trajectories (n=129 observations for 48 individuals)

predictor	*Estimated slope*	*Lower CI*	*Upper CI*	*p-value*

Sex M	-0.00	-0.04	0.02	0.65
Phase - incubation	-0.07	-0.10	-0.05	<0.01***
Phase - rearing	-0.09	-0.12	-0.07	<0.01***
Sex M : incubation	-0.01	-0.04	0.02	0.51
Sex M : rearing	0.02	-0.01	0.05	0.24

*: p-value between 0.05 and 0.01, **: p-value between 0.01 and 0.001, ***: p-value < 0.001, . : p-value between 0.05 and 0.1
