# Supplementary material for: Both movements and breeding performance are affected by individual experience in the Bonelli's eagle Aquila fasciata
Source: Ecol Evol. 2024 Jul 24;14(7):e70081. doi: 10.1002/ece3.70081 (PMC11268896; doi:10.1002/ece3.70081)
Supplement: Supplementary file 6 — Appendix S6 [file ECE3-14-e70081-s006.pdf]

# Both movements and breeding performance are affected by individual experience in the Bonelli's eagle *Aquila fasciata*

Lise Viollat, Alexandre Millon, Cécile Ponchon, Alain Ravayrol, Thibaut Couturier, Aurélien Besnard

APPENDIX S6: Individuals GPS equipment information

Table S6-1: GPS equipment information for Bonelli's eagles of the French population equipped with GPS tags, whose data were used in our analysis, with the breeding site (Site), the sex (M=male, F=female), the GPS tag manufacturer, the year the individual has been equipped with the GPS tag (Equipment year), the breeding years where we have data along all the breeding seasons, the number of GPS locations, the number of days tracked since equipped, and percentage of the GPS tag weight (included the GPS tag and the Teflon harness) compared to the individuals bird mass (% mass).

Individual	Site	Sex	GPS tag manufacturer	Equipment year	Breeding years	Number of GPS locations	Number of days tracked	% Mass
1	16	M	Technosmart	2009	2010	6066	181	3.15
2	33	M	Microwave Argos	2009		6394	1078	3
3	34	F	Microwave Argos	2009		217	63	2.5
4	19	M	Microwave Argos	2011		11180	2037	2.85
5	20	F	Microwave Argos	2011		1635	290	2.5
6	37	M	Microwave Argos	2011		8455	1492	3.12
7	26	M	Microwave Argos	2012		4216	741	3.15
9	20	F	Microwave Argos	2013		1932	343	2.24
11	14	F	Ecotone GSM/UHF	2014	2014	2471	230	1.29
12	21	F	Ecotone GSM/UHF	2014	2015-2016	47943	357	1.23
13	21	M	Ecotone GSM/UHF	2014		7200	342	
14	31	M	Ecotone GSM/UHF	2014	2015	16572	760	
15	35	M	Ecotone GSM/UHF	2014	2015	9259	275	1.95
16	36	M	Ecotone GSM/UHF	2014		2938	312	
17	1	F	Microwave GSM	2015		10424	326	2.36
18	13	M	Ecotone GSM/UHF	2015	2015	17902	580	2.05
19	16	F	Microwave GSM	2015		31131	1186	2.32
21	20	F	Ecotone GSM/UHF	2015	2016-2017-2018-2019	21875	1148	1.29
22	22	M	Ecotone GSM/UHF	2015		22999	1539	1.77
23	24	F	Ecotone GSM/UHF	2015		4492	234	1.45
24	25	F	Ecotone GSM/UHF	2015		16856	709	1.38
25	36	F	Ecotone GSM/UHF	2015	2016-2017	69105	1584	1.44
26	4	M	E-obs GSM/GRS	2016	2017-2018-2019-2020	2319	100	3.38
27	6	M	Ecotone GSM/UHF	2016	2016	87347	1864	1.77
28	25	F	Ecotone GSM/UHF	2016		26158	2286	1.63

29	27	F	E-obs GSM/GRS	2016	2017-2018-2019-2020-2021-2022	80675	1871	
30	33	F	E-obs GSM/GRS	2016	2017	20436	466	2.34
31	87	F	E-obs GSM/GRS	2016	2018-2019-2020-2021	84249	2187	2.72
32	2	M	Ornitela GSM/GPRS	2017	2018-2019	36337	1384	2.29
34	15	M	E-obs GSM/GRS	2017	2019-2020-2021-2022	22193	554	3.3
35	29	M	Microwave GSM	2017	2018-2019-2020-2021	37703	809	3.32
36	35	M	Ornitela GSM/GPRS	2017	2018-2019	121863	2363	2
37	72	F	E-obs GSM/GRS	2017	2019-2020-2021-2022	2630	108	2.72
38	8	F	Ornitela GSM/GPRS	2018	2019	46017	841	2.04
39	10	M	Ornitela GSM/GPRS	2018	2020-2021-2022	39352	1071	2.82
40	86	M	Ornitela GSM/GPRS	2018	2019-2020	29990	592	2.29
41	28	M	Ornitela GSM/GPRS	2019	2019-2020-2021-2022	77631	1802	2.18
42	33	F	E-obs GSM/GRS	2019	2020	67784	1645	3.4
43	75	M	Ornitela GSM/GPRS	2019	2020-2021-2022	15988	316	2.67
44	16	M	Ornitela GSM/GPRS	2020	2021	47895	1126	2.4
45	10	M	Ornitela GSM/GPRS	2021	2022	42554	909	2.44
46	89	M	Ornitela GSM/GPRS	2021	2022	16419	362	2.46
47	5	F	Ornitela GSM/GPRS	2021	2022	31480	683	
